# Changes in oral, skin, and gut microbiota in children with atopic dermatitis: a case-control study

**DOI:** 10.3389/fmicb.2024.1442126

**Published:** 2024-08-15

**Authors:** Xueer Zhang, Xiaomin Huang, Pai Zheng, E. Liu, Sixian Bai, Shuoyu Chen, Yaobin Pang, Xinyu Xiao, Huifang Yang, Jing Guo

**Affiliations:** ^1^Department of Dermatology, Chengdu University of Traditional Chinese Medicine, Chengdu, China; ^2^Department of Dermatology, Hospital of Chengdu University of Traditional Chinese Medicine, Chengdu, China

**Keywords:** inflammatory skin diseases, atopic dermatitis, skin microbiota, oral microbiota, gut microbiota

## Abstract

**Introduction:**

Atopic dermatitis (AD) is a common clinical recurrent atopic disease in dermatology, most seen in children and adolescents. In recent years, AD has been found to be closely associated with microbial communities.

**Methods:**

To explore the synergistic effects between colonizing bacteria from different sites and AD, we comparatively analyzed the skin, oral, and gut microbiota of children with AD (50 individuals) and healthy children (50 individuals) by 16S rRNA gene sequencing. Twenty samples were also randomly selected from both groups for metabolic and macrogenomic sequencing.

**Results:**

The results of our sequencing study showed reduced microbiota diversity in the oral, skin, and gut of children with AD (*P* < 0.05). Metabolomics analysis showed that serotonergic synapse, arachidonic acid metabolism, and steroid biosynthesis were downregulated at all three loci in the oral, skin, and gut of children with AD (*P* < 0.05). Macrogenomic sequencing analysis showed that KEGG functional pathways of the three site flora were involved in oxidative phosphorylation, ubiquitin-mediated proteolysis, mRNA surveillance pathway, ribosome biogenesis in eukaryotes, proteasome, basal transcription factors, peroxisome, MAPK signaling pathway, mitophagy, fatty acid elongation, and so on (*P* < 0.05).

**Discussion:**

The combined microbial, metabolic, and macrogenetic analyses identified key bacteria, metabolites, and pathogenic pathways that may be associated with AD development. We provides a more comprehensive and in-depth understanding of the role of the microbiota at different sites in AD patients, pointing to new directions for future diagnosis, treatment and prognosis.

## 1 Introduction

Atopic dermatitis is the most common skin disease, it is a recurrent, dermatosis with a defective skin barrier, Th2 inflammatory profile, allergic tendency, and dysbiosis. The condition predominantly affects infants and adolescents, though it can persist into adulthood or old age. The estimated prevalence rate in children and adults is approximately 15%−20% and 1%−3%, respectively, with an upward trend observed in recent decades (Kim et al., 2019). Children with AD often present comorbidities with other allergic conditions such as food allergy, asthma, and allergic rhinitis, collectively known as the atopic triad (Hill and Spergel, 2018). The pathogenesis of AD involves environmental factors, genetic predispositions, skin barrier dysfunction, and immune system defects (Edslev et al., 2020; Masuka et al., 2020).

Most microbiome studies of inflammatory skin diseases have utilized 16S sequencing to specify microbial alterations in dermatoses, elucidating important findings. However, deciphering only bacterial genera and their relative abundance has had limited impact on improving clinical applications (Chen et al., 2023). In recent years, with the application of various technologies such as metatranscriptomics, macrogenomics, and metabolomics, the important role of microbes in the pathogenesis of AD has been revealed, as well as the demonstration of relationships that characterize the interactions of proteins and small molecules consumed and produced by microorganisms, providing a greater understanding of the underlying mechanisms of how microbes interact with the immune system.

There is growing evidence that dysbiosis of the microbiome localized to a disease can have an associated pathological impact on distant microbes, referred to as the “skin-gut” or “skin-brain” axis. This also suggests that the microbiomes of our different body parts may interact in the development of AD. Studies have shown that since the oral cavity and the gastrointestinal tract are interconnected components of the digestive system, oral bacteria can ectopically colonize the gut and thus participate in the development of the disease (Flemer et al., 2018). Skin and gut cells originate from the same embryonic layer and share similar signaling and innervation pathways (Park et al., 2021). Furthermore, skin and gut function as immune barriers, exhibit biological dimorphism and play similar roles (Mahmud et al., 2022). Although the exact mechanisms are unknown, a comprehensive study of multiple microbiotas at different sites may provide insight into the link between disease, microbiota, and host. We aimed to analyze the distribution of oral, skin, and gut microbiota in children with AD, to explore the significance of each site's microbiota in the pathogenesis of AD, and to lay the groundwork for further studies on the relationship between the colonizing microbiota at different sites.

## 2 Methods

### 2.1 Study design

**Figure 1:**
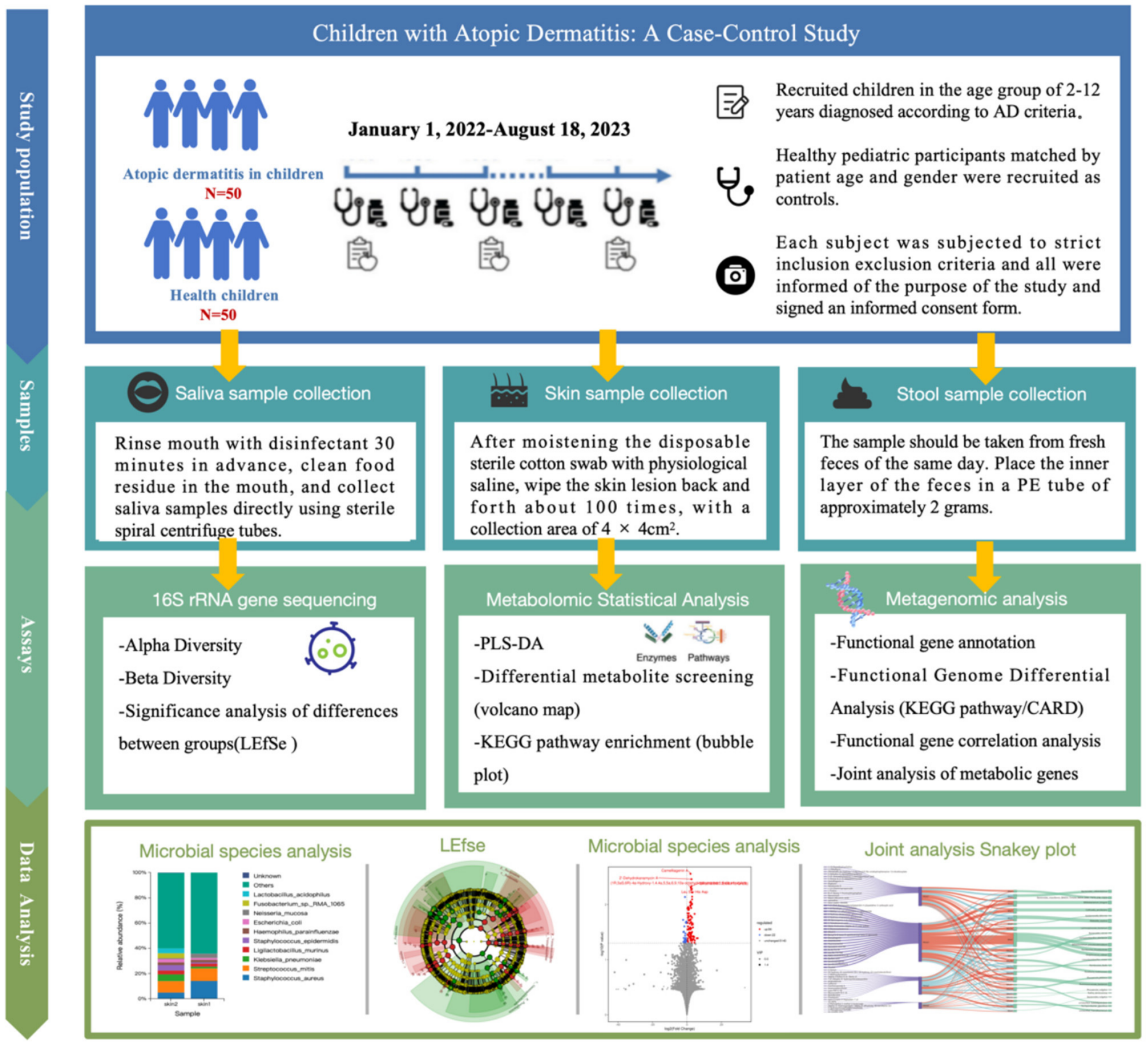
Changes in oral, skin, and gut microbiota in children with atopic dermatitis: a case-control study.

The protocol of this study was approved by the Research Ethics Committee of the Affiliated Hospital of Chengdu University of Traditional Chinese Medicine (Chengdu, Sichuan, China) (Ethical code: 2022KL-059). Each participant was informed of the purpose of the study and signed an informed consent form. The personal data of each participant were kept confidential. We designed a cross-sectional study (January 1, 2022–August 18, 2023) at the Dermatology Clinic of the Affiliated Hospital of Chengdu University of Traditional Chinese Medicine. A cohort of 100 individuals was analyzed. We recruited children in the age group of 2–12 years (*n* = 50) diagnosed according to AD criteria (Sroka-Tomaszewska and Trzeciak, 2021). Healthy pediatric participants (*n* = 50) matched by patient age and gender were recruited as controls (Children who went to the hospital for healthy children). Exclusion criteria for both the case and control groups were children with primary diseases such as hepatic or renal insufficiency, hematopoietic insufficiency, or physiological defects; children with other skin diseases in combination with the skin lesions; children with combined medical illnesses such as fever, cough, diarrhea, and constipation; any type of oral disease; and the use of antibiotics or the use of probiotics in the past 3 months. To address potential sources of bias, confounding factors were considered in our exclusion criteria (Claesson et al., 2017) (Figure 1).

### 2.2 Sample collection

(1) The principle that microbial sequencing should incorporate an adequate sample size was followed, combined with the susceptibility of the samples to environmental, individual, and other factors. A randomized numeric table method was used to select 50 subjects in each of the healthy and diseased groups, and skin, saliva, and stool samples were collected during visits to the clinic, and the names, ages, and groups of the samples were recorded in detail. (2) Subjects signed an informed consent form and were instructed to avoid the use of hand sanitizers and antibacterial soaps as well as skin care products for 1 week and not to shower for the first 24 h. Avoid eating, drinking, and performing any oral hygiene maneuvers for at least 3 h. Prepare sterile swabs, gloves, markers, and labeling stickers. (3) Sample collection procedures were as follows: (A) Saliva sample collection: the sample should be taken 30 min beforehand by rinsing mouth with sterilized water to clean up the food residues in the mouth, and after 30 min, the researcher should wear sterile gloves and use the sterile screw-open centrifugal tube to collect saliva directly. (B) Skin sample collection: moisten the disposable sterile swab with saline, then wipe the skin lesions on the children (applying stable pressure and consistent friction), back and forth for about 100 times within 2 min at the sampling point, collecting an area of 4 × 4 cm^2^, and marking the area after collection. (C) Stool sample collection: the children and their families should be instructed to avoid mixing urine with feces when collecting feces and the samples should be taken from fresh feces of the same day, and after removing the outer layer of feces by using the sterile swabs, the inner feces of the inner layer of feces should be placed in the PE tubes of approximately 2 g. (4) Store the specimen in the refrigerator at −80°C for storage within 2 h after sampling is completed. Afterward, the specimens were transported to the laboratory for high-throughput sequencing of bacterial colonies in batches preserved on dry ice.

### 2.3 DNA extraction and 16S rRNA gene sequencing

(1) Extraction of genomic DNA from the samples. The DNA was extracted in strict accordance with the instructions of the DNA extraction kit, and the DNA was quantified by Nanodrop 2,000C spectrophotometer, and the purity and concentration of DNA were detected by agarose gel electro swimming after the completion of DNA extraction. (2) PCR amplification of target fragments. The target fragments were amplified by PCR using 27F (16S-F) (5′-AGRGTTTGATYNTGGCTCAG-3′) as a general primer for the full-length region of 16S rRNA. (3) PCR amplification and product purification. Add dNTPDNA polymerase and other reagents required for PCR reaction, mix, and centrifuge, and then put it into the PCR instrument for PCR amplification. (4) Quantification of concentration (Qubit) and mixing of samples. The PCR products are quantified by Qubit and the concentration of the PCR products is adjusted. (5) Sequencing library preparation. Sequencing libraries were prepared using Illumina's TruSeqNano DNA LT Library Prep Kit. (6) High-throughput sequencing. Before online sequencing, use Agi-lent 2100 bioanalyzer to check the Qubit and size of the library, and use PacBio Binding Kit to bind the library before on-line sequencing; purify the final reaction products with AMpure PB Beads, and then put them on Sequel II sequencer for on-line sequencing.

### 2.4 Microbial bioinformatics analysis

The data from the study were processed, saved, and statistically analyzed using SPSS 26.0 software. A *T*-test was used for data following a normal distribution, while a non-parametric test was used for data that did not. Count data were analyzed with a chi-square test. Alpha and beta diversity indices were compared using the QIIME-1 procedure, and the Linear Discriminant Analysis (LDA) method identified differences in skin, oral, and gut microbiota in AD. Mass spectrometry data underwent multivariate statistical analyses. Principal component analysis (PCA) was first performed to observe differences in metabolic patterns and natural clustering trends between groups, with loading plots used to identify variables contributing to group categorization. Orthogonal partial least squares discriminant analysis (OPLS-DA) was then used to identify differences between the two groups of samples and to screen for differential metabolites (Wen et al., 2017). The screening criteria for differential metabolites were VIP > 1, *P* < 0.05, and FC ≥ 1.5 or FC ≤ 0.7. Differential metabolites were imported into the KEGG database (https://www.genome.jp/kegg) for metabolic pathway enrichment analysis.

Macrogenomic profiles were compared at each phylogenetic level using MetaStats 2.0 software and the Kyoto Encyclopedia of Genes and Genomes (KEGG) functional categories to identify taxa. In the analysis, *p* > 0.05 indicated no statistical significance, while *p* < 0.05 indicated statistical significance.

To further investigate the relationship between colonizing flora at different sites and AD, we compared the skin, oral, and gut microbiota of children with AD (50 individuals) and healthy children (50 individuals) using 16S *rRNA* gene sequencing. DNA extracted from skin, oral, and gutsamples was sequenced for 16S *rRNA* gene amplicon fragments on the Illumina MiSeq platform, with results analyzed via QIME/MetaStats 2.0 software. Additionally, we randomly selected 20 samples from each group for metabolic and metagenomic sequencing. The integration of microbiome, metabolome, and metagenomic data aimed to explore the synergistic role of microbiota in these three body sites.

## 3 Results

### 3.1 Clinical characteristics

The basic characteristics of the AD and healthy groups are shown in Table 1. There was no difference in age, gender, and body mass index (BMI) between the two groups. The age range of the participants was 2 to 12 years. The mean age of onset of migraine patients was 6.58 ± 2.21 years, duration of disease was 3.4 ± 0.744 years, and body weight was 25.10 ± 3.33 Kg.

**Table 1:** The basic characteristics of the AD and healthy groups.

**Characteristics**	**AD group (*n =* 50)**	**Control group (*n =* 50)**
Age (years)^a^	6.58 ± 2.21	6.33 ± 2.25
Female (*n*, %)	23 (57.5%)	21 (52.5%)
Weight (kg)^a^	25.10 ± 3.33	25.75 ± 2.79
Course of disease (years)^a^	3.40 ± 0.744	-

Data are shown as mean (SD). ^a^ By *T-*test, the two groups, AD group and healthy group, *p* > 0.05, the difference between age and weight of the two groups is not statistically significant. By chi-square test for gender in the two groups, χ^2^ = 0.202, *p* > 0.05, the difference was not statistically significant.

### 3.2 Basic information on flora sequencing in children with atopic dermatitis

A total of 50 samples were collected in the patient group, of which (1) 50 oral samples were sequenced successfully, obtaining 366,528 high-quality reads and 1,756 OTUs; and the taxonomic information corresponding to each OUT was obtained through Silva database matching. (2) Six skin samples did not produce sufficient DNA, 44 samples were sequenced successfully, obtaining 190,697 high-quality reads and 3,657 OTUs; (3) 3 stool samples did not produce sufficient DNA, 47 samples were sequenced successfully, obtaining 226,831 high-quality reads and 2,241 OTUs. In the control group, a total of 50 samples were collected, of which (1) 50 oral samples were sequenced successfully, obtaining 322,066 high-quality reads and 2,630 OTUs; Each OUT was compared with the Silva database to obtain the corresponding taxonomic information; (2) 4 skin samples did not produce sufficient amount of DNA, 36 skin samples were sequenced successfully, obtaining 192,049 high-quality reads and 3,605 OTUs; (2) 50 stool samples were sequenced successfully, obtaining 239,764 high-quality reads and 2,443 OTUs.

### 3.3 Overall distribution of flora in children in the patient and control groups

**Figure 2:**
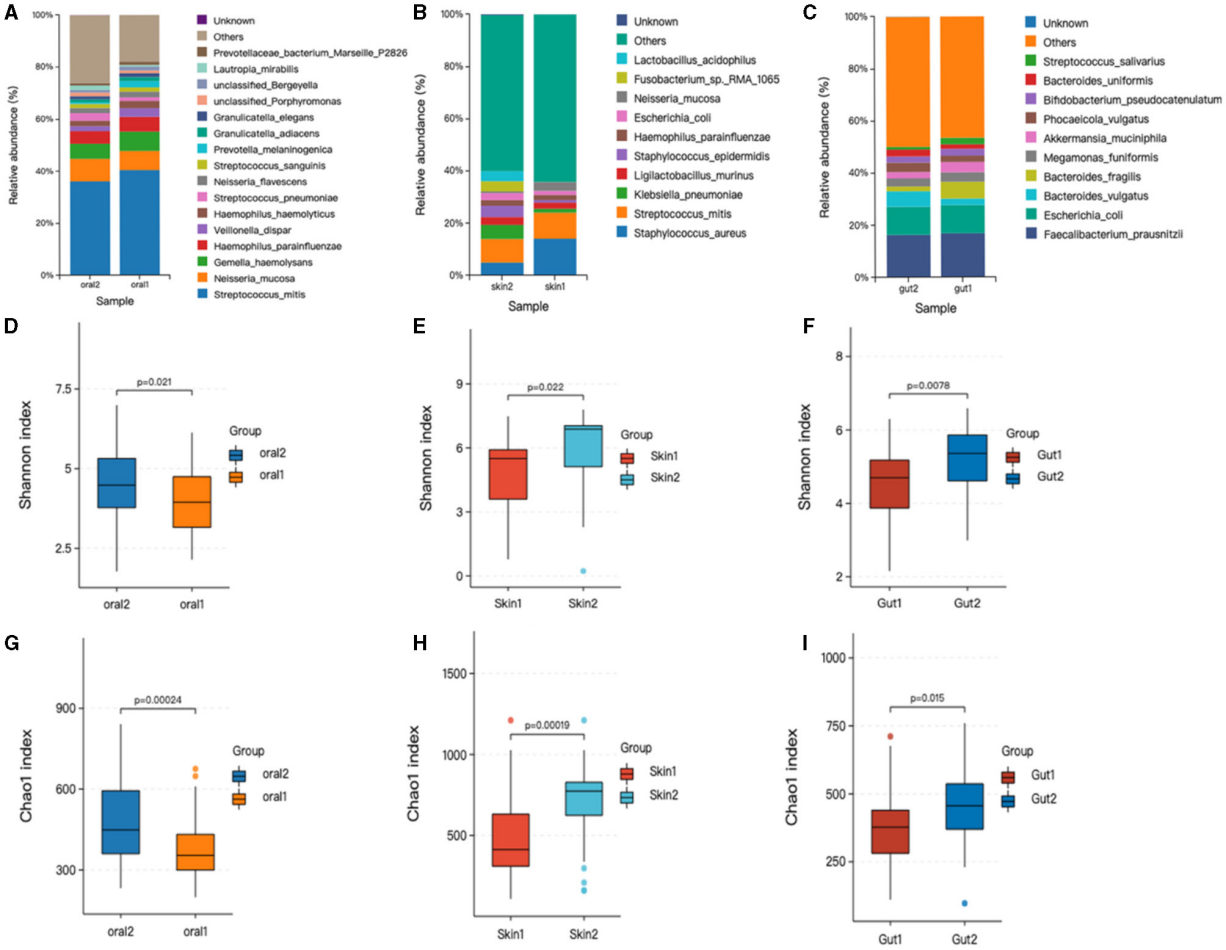
Changes in oral, skin, and gut bacterial species and diversity in children with AD. skin1/oral1/gut1: AD sample group; skin2/oral2/gut2: control sample group. The symbol * represents data points other than the minimum and maximum values. **(A)** Histogram of horizontal distribution of oral flora species; **(B)** Histogram of horizontal distribution of skin flora species; **(C)** Histogram of horizontal distribution of intestinal flora species; **(D)** Shannon index of oral flora patient group and control group; **(E)** Shannon index of skin flora patient group and control group; **(F)** Shannon index of gutl flora patient group and control group; **(G)** Oral flora patient group and control group chao1 index; **(H)** Skin strain patient group vs. control group chao1 index; **(I)** Gut strain patient group vs. control group chao1 index.

Relative abundance of skin, oral, and gut microbiota in AD and control groups. Bar graphs show the relative abundance of oral microbiota at the species level. (1) Oral microorganisms were detected in the patient group, the distribution of which is shown in Figure 2A. The major taxa and proportions were (The relative abundance of bacterial strains accounts >5%): *Streptococcus_mitis (S. mitis)* 40%, *Neisseria_mucosa (N. mucosa)* 7.3%, *Gemella_haemolysans (G. haemolysans)* 7.4%, *Haemophilus_parainfluenzae (H. parainfluenzae)* 5.6%. The major taxa and proportions of the control group were *S. mitis* 36%, *N. mucosa* 8.6%, and *G. haemolysans* 5.7%. (2) Skin microorganisms were detected in the patient group, the distribution of which is shown in Figure 2B. The major taxa and proportions were (The relative abundance of bacterial strains accounts >5%): *Staphylococcus_aureus (S. aureus) 14%, S. mitis 10%*. The major taxa and proportions of the control group were *S. aureus 7.1%, and S. mitis 5.9%*. (3) Gut microorganisms were detected in the patient group, the distribution of which is shown in Figure 2C. The major taxa and proportions were (The relative abundance of bacterial strains accounts >5%): *Faecalibacterium_prausnit (F. prausnit) 17.2%, Escherichia_coli (E. coli) 10.5%*, and *Bacteroides_fragilis 6.4%*. The major taxa and proportions of the control group were *F. prausnit 19.9% and E. coli 8.5%*.

### 3.4 Alpha diversity

Based on the OTU level, the Chao and Shannon indices were used to measure sample species richness and species diversity (Finn, 2024). We observed that the Shannon and Chao1 indices for the oral, skin, and gut microbiota of children with AD were significantly lower than those of the control group (Figure 2). This indicates that the richness and diversity of the microbiota in children with AD were markedly reduced compared to healthy children. Additionally, we compared the diversity indices of the microbiota before and after treatment within the AD group using the Mann-Whitney U rank-sum test. The results showed statistically significant differences in both the Chao1 index (*P* < 0.001) and the Shannon index (*P* < 0.001) between the two groups.

### 3.5 Beta diversity

**Figure 3:**
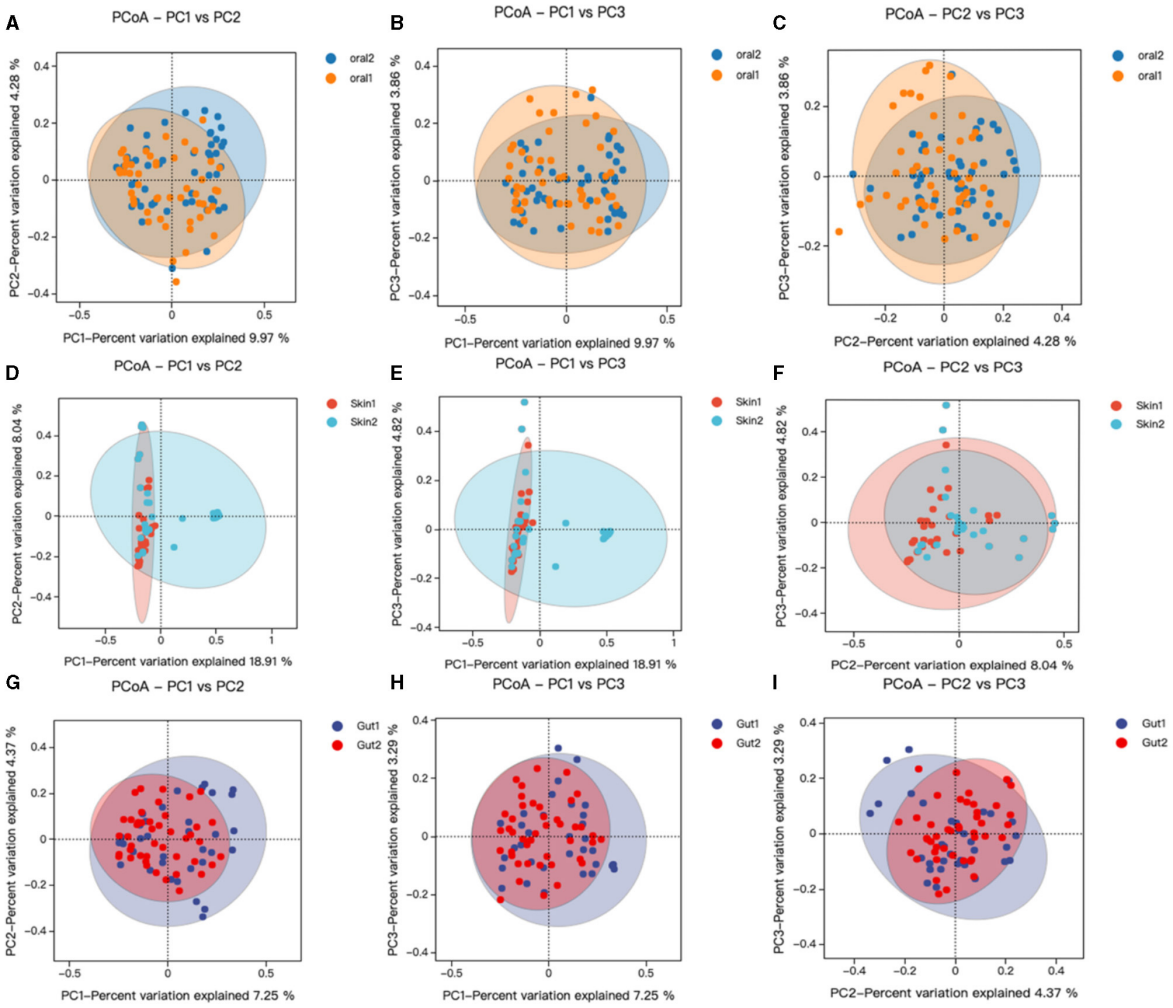
Changes in oral, skin, and gut bacterial beta diversity in children with AD. skin1/oral1/gut1: AD sample group; skin2/oral2/gut2: control sample group. We analyzed the beta diversity of the AD microbiota and controls by principal coordinate analysis of weighted UniFrac distances, and then plotted PCoA analyses using R language tools out. Similarity between samples was indicated by the distance between samples spanning the three principal coordinates (PC 1, PC 2, and PC 3), with the distance between samples indicating the degree of similarity between them. **(A–C)** PCoA analysis of oral strains in patient and control groups; **(D–F)** PCoA analysis of skin strains in patient and control groups; **(G–I)** PCoA analysis of gut strains in patient and control groups.

Beta diversity analysis was performed using QIIME software to compare the degree of similarity in species diversity present in different samples (Gail et al., 2021). The similarity analysis showed that there was a significant difference in bacterial community composition between skin, oral, and gut microorganisms, and controls among children with AD (*P* < 0.05). (1) There was a significant difference in bacterial community composition between oral microbes and controls (R = −0.05, *p* = 0.006) (Figures 3A–C); (2) there was a significant difference in bacterial community composition between skin microbes and controls (R = 0.28, *p* = 0.001) (Figures 3D–F); and (3) there was a significant difference in bacterial community composition between gut microbes and controls (R = 0.94, *p* = 0.001) (Figures 3G–I).

### 3.6 Analysis of community structure by group

#### 3.6.1 Significance analysis of differences in community structure between groups

**Figure 4:**
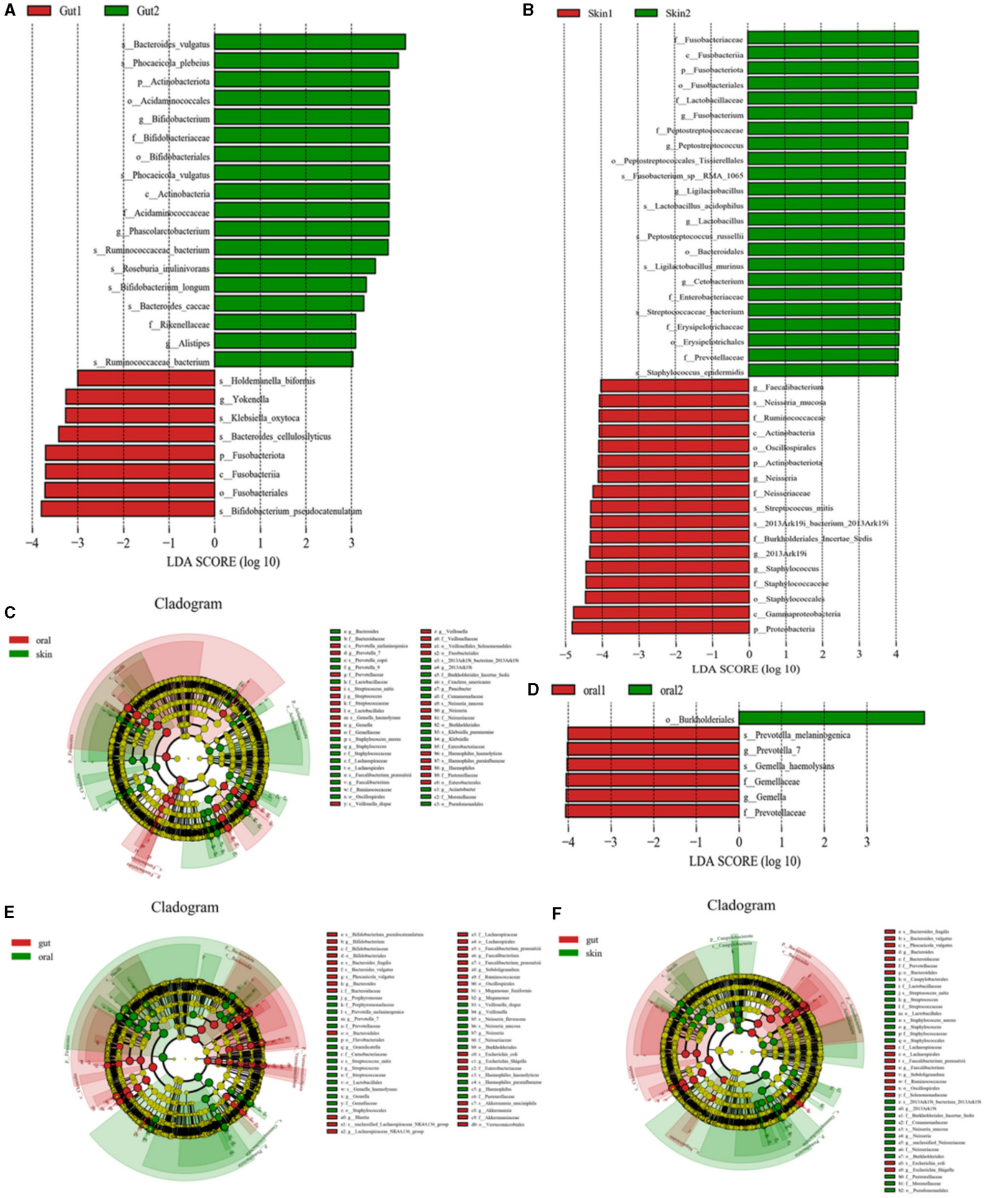
Significant analysis of bacterial species differences among oral, skin, and gut populations in children with AD. **(A)** LEfse plot of gut strains in patient group and experimental group; **(B)** LEfse plot of skin strains in patient group and experimental group; **(C)** LEfse plot of oral vs. skin strains in patient group and experimental group; **(D)** LEfse plot of oral strains in patient group and experimental group; **(E)** LEfse plot of oral vs. gut strains in patient group and experimental group; **(F)** LEfse plot of skin vs. gut strains in patient group and experimental group.

Species levels in the three major microbiotas analyzed by LEfSe showed significant changes between the patient and control groups. (1) Oral microorganisms were detected in the patient group, and their distribution is shown in Figure 4D. Including the following microbiota: *Prevotella_melaninogenica, G. haemolysans, E. coli, among others*. (2) Skin microorganisms were detected in the patient group, and their distribution is shown in Figure 4B. Including the following microbiota: *N. mucosa, Faecalibacterium, and S. mitis, among others*. (3) Gut microorganisms were detected in the patient group, and their distribution is shown in Figure 4A. Including the following main microbiota: *Klebsiella_oxytoca, Romboutsia_ilealis, and Streptococcus_intermedius, among others*.

To further confirm the variability of the flora in three different sites, the skin, the oral cavity, and the gut, we compared the species levels of the three main microbiotas using LEfSe analysis. (1) We found that the main differential bacteria in the oral cavity and skin tract were predominantly at the genus level: *Bacteroides, Prevotella_7, Streptococcus, Gemella, Staphylococcus, Faecalibacterium, Veillonella,2013Ark19i, Paucibacter, Neisseria, Klebsiella, Haemophilus*, and *Acinetobacter*; (Figure 4C) (2) we found that the main differential bacteria in the oral cavity and gut tract were predominantly at the genus level: *Bifidobacterium, Bacteroides, Porphyromonas, Prevotella_7, Granulicatella, Streptococcus, Gemella, Blautia, Lachnospiraceae_NK4A136_group, Faecalibacterium, Subdoligranulum, Megamonas, Veillonella, Neisseria, Escherichia, Shigella, Haemophilus, Akkermansia*; (Figure 4E) (3) the main differential bacteria in the gut and skin are: *Bacteroides, Streptococcus, Staphylococcus, Faecalibacterium, Subdoligranulum, 2013Ark19i, Neisseria, unclassified_Neisseriaceae, Escherichia_Shigella* (Figure 4F) (Specific information can be found in Supplementary Tables 1–3).

#### 3.6.2 Similarity analysis of community structure across groups

**Figure 5:**
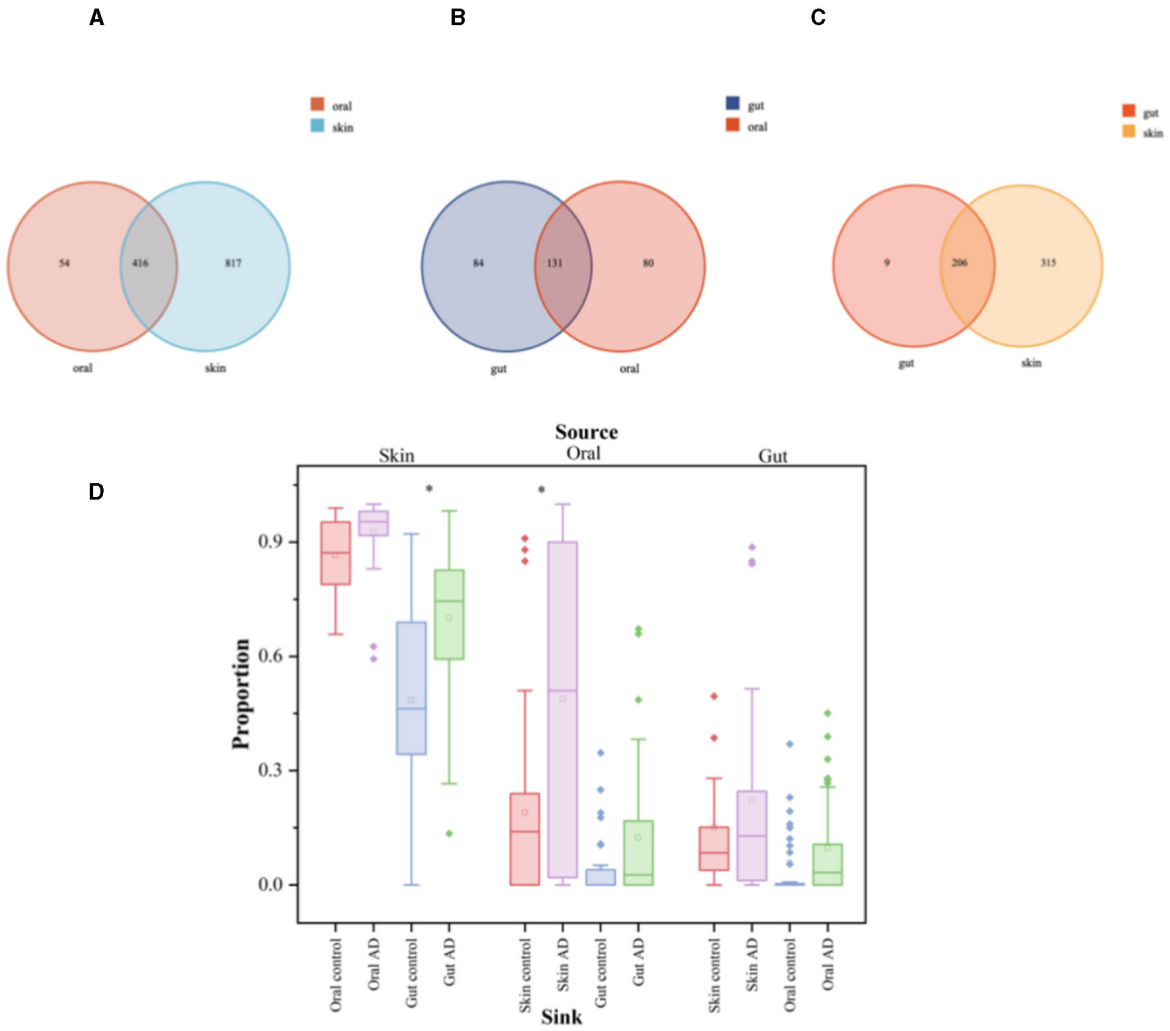
Significant analysis of bacterial species differences among oral, skin, and gut populations in children with AD. The symbol * represents data points other than the minimum and maximum values. **(A)** Venn's diagram of oral and skin co-strains; **(B)** Venn's diagram of oral and gut co-strains; **(C)** Venn's diagram of skin and gut co-strains; **(D)** Diagram of the source analysis of the bacterial flora in patients with AD.

Oral, skin, and gut microorganisms of children with AD were analyzed for intergroup similarity and represented by Wayne plots. There are 416 similar bacteria in the skin and oral cavity, of which the major species are: *S. mitis, G. haemolysans, N. mucosa, H. parainfluenzae, Streptococcus_sanguinis, Granulicatella_adiacens, among others* (Figure 5A). There are 131 similar bacteria in the oral and gut, of which the major species are: *Streptococcus_salivarius (S. salivarius), H. parainfluenzae, Veillonella_parvula, Granulicatella_adiacens, Candidatus_Nanosynbacter_lyticus, S. mitis, among others* (Figure 5B). There are 206 similar bacteria in the gut and skin, of which the major species are: *F. prausnit, S. aureus, S. mitis, 2013Ark19i_bacterium_2013Ark19i, Escherichia_coli, S. salivarius, among others* (Figure 5C). The results are shown in Supplementary Tables 4–6. We performed microbiota tracing analysis and observed that when the oral sample was used as the source and the skin sample as the target, the skin and oral microbiota in children with AD showed a greater similarity compared to the control group. Similarly, when the skin sample was used as the source and the gut sample as the target, AD children's skin and gut microbiota showed higher similarity compared to the control group. Conversely, when the gut sample was the source and skin and oral samples were the targets, there was no significant difference in similarity between AD children's gut microbiota and the skin and oral microbiota compared to the control group. Our data indicate that compared to the control group, AD children's skin microbiota is more like their oral and gut microbiota, suggesting that a considerable portion of the skin microbiota may originate from the oral cavity, while the gut microbiota may stem from the skin (Figure 5D).

The box plots illustrate the predicted proportion of AD children and control groups in which one body site originates from another body site: using skin samples as the “source” and oral or gut samples as the “target” (left); using oral samples as the “source” and skin or gut samples as the “target” (center); using gut samples as the “source” and skin or oral samples as the “target” (right). Two-tailed Wilcoxon rank-sum test was used to determine significance. The box plots show the median and interquartile range determined by the Tukey method; black dots represent data points outside the minimum and maximum values.

### 3.7 Metabolite information analysis

In this study, 20 oral, skin, and gut microbial samples were randomly selected for metabolic analysis in each of the patient and control groups. Raw data collected using MassLynx V4.2 were processed using Progenesis QI software for peak extraction, alignment, and other data processing operations. A total of 7,723 metabolites were obtained, with 4,692 in positive ion mode and 3,031 in negative ion mode. The Pearson correlation coefficients between QC samples were calculated based on the relative quantitative values of metabolites. The results showed that, in the positive ion mode, the correlation coefficients between QC samples were all >0.98, and in the negative ion mode, they all reached 0.99. This indicates good data quality, meeting the requirements for subsequent experiments.

#### 3.7.1 Metabolomics analysis-based observation of species diversity of oral, skin, and gut microbiota

**Figure 6:**
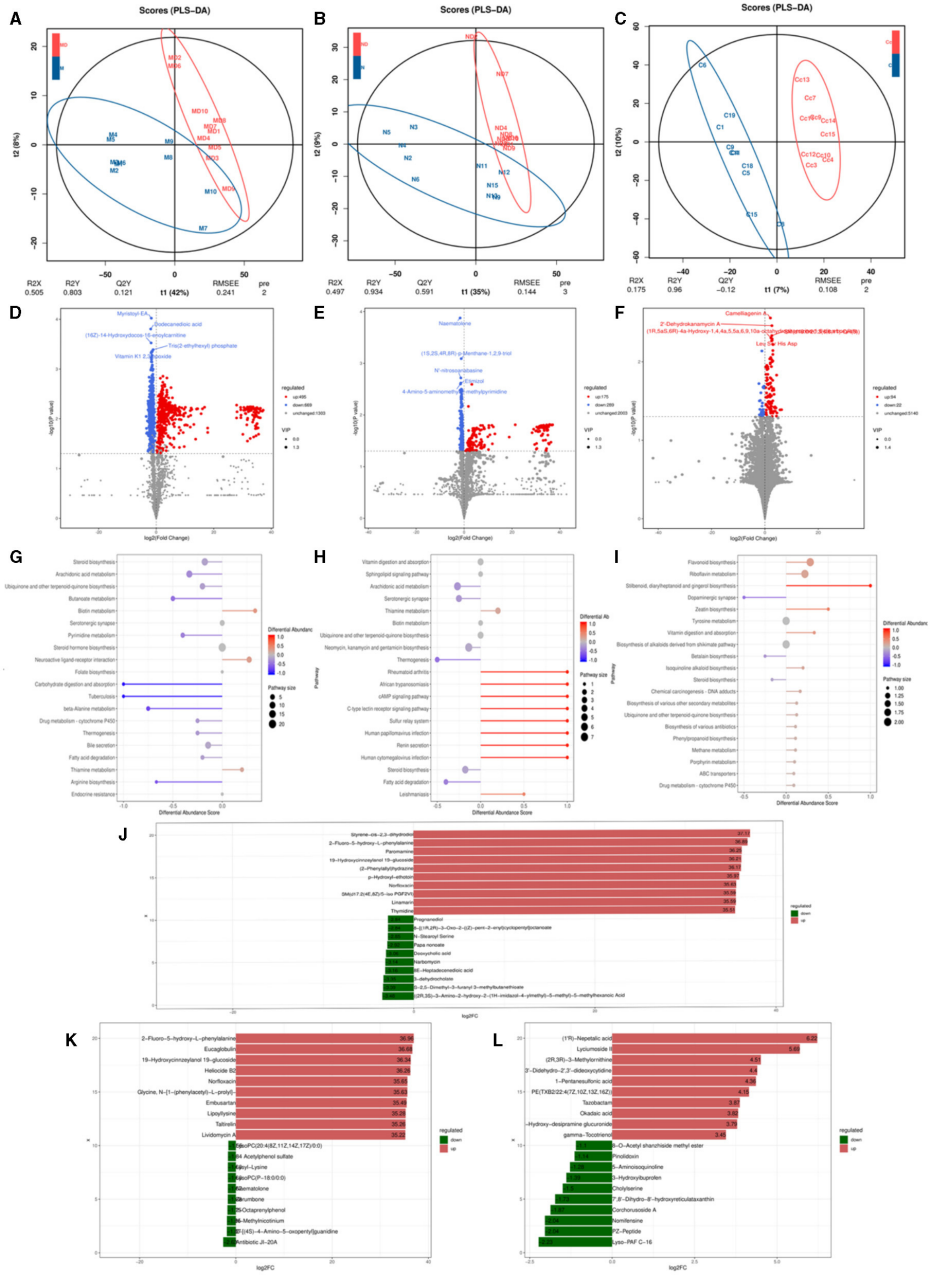
Metabolomics analysis based on observation of species diversity of oral, skin, and gut microbiota. **(A)** Oral metabolite OPLS-DA analysis plot; **(B)** Skin metabolite OPLS-DA analysis plot; **(C)** Gut metabolite OPLS-DA analysis plot; **(D)** Oral differential metabolite volcano plot; **(E)** Skin differential metabolite volcano plot; **(F)** Gut differential metabolite volcano plot; **(G)** Oral differential metabolite KEGG abundance plot; **(H)** Skin differential metabolite KEGG abundance plot; **(I)** Gut differential metabolite KEGG abundance map; **(J)** Oral differential metabolite LEfse map; **(K)** Skin differential metabolite LEfse map; **(L)** Gut differential metabolite LEfse map.

OPLS-DA analysis was conducted on pediatric AD subjects in the patientand control groups at three different sites: oral, skin, and gut. The goodness of fit and predictive ability values indicated that the OPLS-DA model possessed excellent predictive capabilities, revealing significant differences between the two groups [for the oral site: R2X = 0.537, R2Y = 0.732, Q2 = 0.15, *P* < 0.005 (Figure 6A); skin site: R2X = 0.732, R2Y = 0.995, Q2 = 0.818, *P* < 0.005 (Figure 6B); and gut site: R2X = 0.174, R2Y = 0.925, Q2 = 0.047, *P* < 0.005 (Figure 6C)]. Metabolites with fold changes ≥2 or ≤ 0.5 and determined through Student's *t*-test analysis were selected as differentially expressed metabolites (*P* < 0.05). Within the patientand control groups, the oral site exhibited 1,164 significantly different metabolites predicted as potential biomarkers for pediatric AD. Of these 1,164 differentially expressed metabolites, 495 were up-regulated and 669 were down-regulated, with the most significantly different metabolite being myristoyl-EA, dodecanedioic acid, (16Z)-14-Hydroxydocos-16-enoylcarnitine, tris(2-ethylhexyl) phosphate, vitamin K1 2,3 epoxide, as shown in Figure 6D. The skin site revealed 464 significantly different metabolites predicted as potential biomarkers for pediatric AD. Among these 464 differentially expressed metabolites, 175 were upregulated, and 289 were downregulated, with the most significantly different metabolite being naematolone, (1S, 2S, 4R, 8R)-p-Menthane-1,2,9-triol, N'-nitroso anabasine, etimizol, 4-Amino-5-aminomethyh2-methylpyrimidine, as shown in Figure 6E. The gut site displayed 116 significantly different metabolites predicted as potential biomarkers for pediatric AD. Among these 116 differentially expressed metabolites, 94 were upregulated, and 22 were downregulated, with the most significantly different metabolite being camelliagenin A, 2′-Dehydrokanamycin A, Le S His Asp, as shown in Figure 6F.

In the metabolic pathways synthesized in AD, various metabolites are enriched. Specifically, in the oral site, the upregulated metabolite is Neuroactive ligand-receptor interaction, while the downregulated metabolites include steroid biosynthesis, arachidonic acid metabolism, ubiquinone, and other terpenoid-quinone biosynthesis, bile secretion, steroid hormone biosynthesis, serotonergic synapse, fatty acid biosynthesis - glutathione metabolism, retrograde endocannabinoid signaling, among other metabolic pathways (Figure 6G). In the skin site, the upregulated metabolite is vitamin B6 metabolism, and the downregulated metabolites include sphingolipid signaling pathway vitamin digestion and absorption, ubiquinone and other terpenoid-quinone biosynthesis, arachidonic acid metabolism, neomycin, kanamycin and gentamicin biosynthesis, steroid biosynthesis, neuroactive ligand-receptor interaction, cutin, alpha-Linolenic acid metabolism, diabetic cardiomyopathy, drug metabolism–cytochrome P450, among other metabolic pathways (Figure 6H). In the gut site, the upregulated metabolites are riboflavin metabolism, ABC transporters, chemical carcinogenesis of DNA adducts, drug metabolism by cytochrome P450, biosynthesis of various other secondary metabolites, biosynthesis of various antibiotics, and cytochrome P450 metabolism of exogenous substances, and the downregulated metabolites include tyrosine metabolism, steroid biosynthesis (Figure 6I). After qualitative and quantitative analysis of the detected metabolites, the diversity of differences in quantitative information between the patientand control groups was compared. Compared to the control group, the logFC results of the top 10 metabolites in the oral (Figure 6J), skin (Figure 6K), and intestinal (Figure 6L) sites of the patient group were upregulated and downregulated.

**Figure 7:**
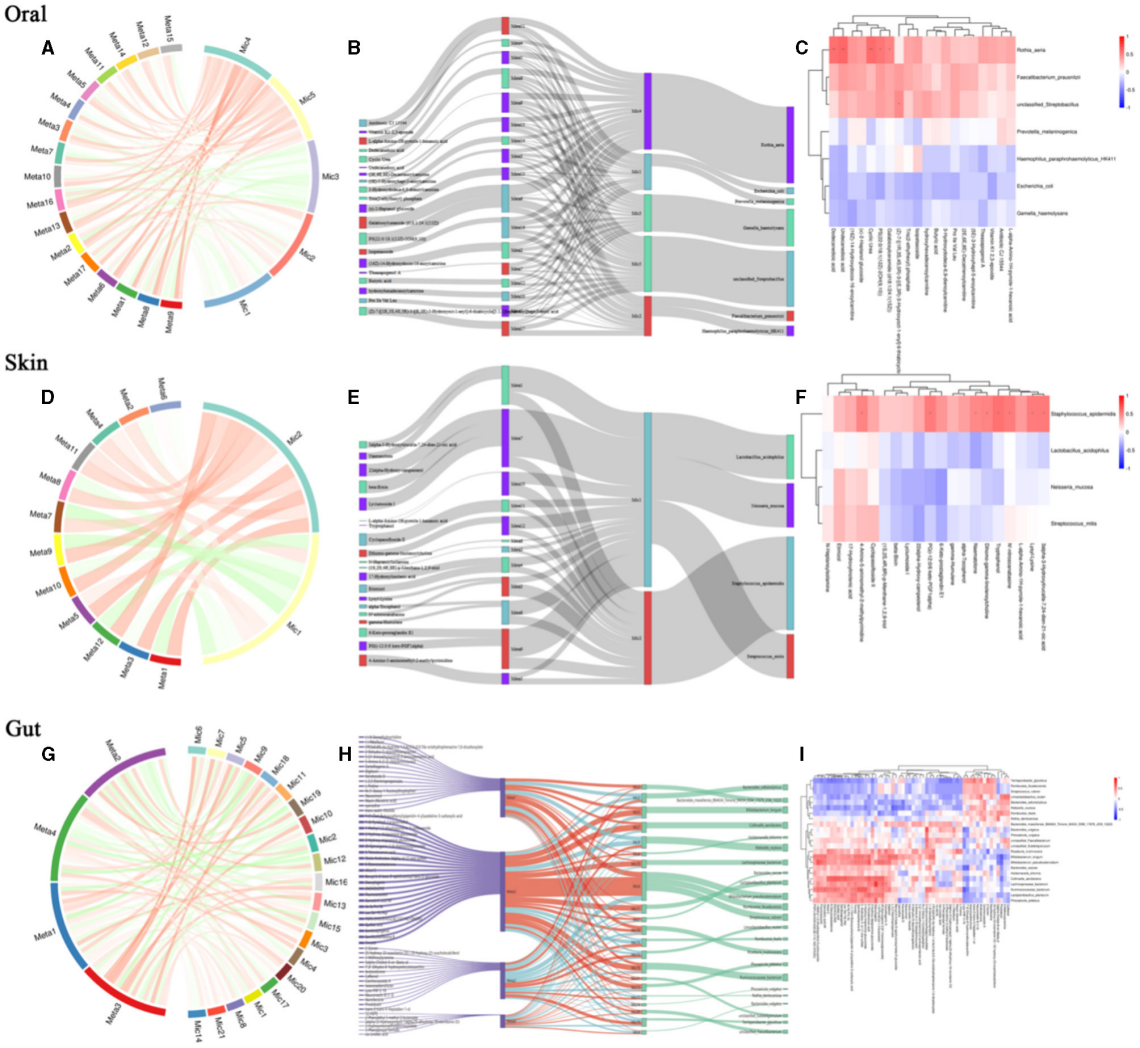
Relationship between differential microbial strains and metabolites. **(A)** String diagram of oral differential strains and differential metabolites; **(B)** Sankey diagram of oral differential strains and differential metabolites; **(C)** Heat map of oral differential strains and differential metabolites; **(D)** String diagram of skin differential strains and differential metabolites; **(E)** Sankey diagram of skin differential strains and differential metabolites; **(F)** Heat map of skin differential strains and differential metabolites; **(G)** String diagram of gut differential strains and differential metabolites; **(H)** Sankey diagram of gut differential strains vs. differential metabolites; **(I)** Heat map of gut differential strains vs. differential metabolites.

To further explore the intrinsic interactions between metabolites and microbial communities, we conducted additional investigations into the correlations between metabolites (ex20) in the oral, skin, and fecal samples and the three microbial communities (ex20). The Sankey diagram depicts significant relationships between the gut metabolite modules and gut microbial modules (*P* < 0.05). Colors indicate the direction of association (red: positive; blue: negative). The relationship between oral microbiota and oral metabolites is as follows: Significant relationships between *Rothia_aeria* and the metabolite undecanedioic acid, cyclic urea, dodecanedioic acid, galabiosylceramide [d18:1/24:1(15Z)] (R = 0.59, *P* = 0.005; R = 0.57 *P* = 0.007; R = 0.56, *P* = 0.009; R = 0.48, *P* = 0.028) (Figures 7A–C). The relationship between skin microbiota and skin metabolites is as follows: *Staphylococcus_epidermidis* and the metabolite tryptophanol, 3alpha-3-Hydroxytirucalla-7,24-dien-21-oic acid, N'-nitroso anabasine, lysyl-Lysine, PG(i-12:0/6 keto-PGF1alpha), dihomo-gamma-linolenoylcholine,4-Amino-5-aminomethyl-2-methyl pyrimidine, naematolone (R = 0.56, *P* = 0.011; R = 0.53, *P* = 0.017; R = 0.52, *P* = 0.019; R = 0.52, *P* = 0.020; R = 0.51, *P* = 0.021; R = 0.49, *P* = 0.029; R = 0.49, *P* = 0.030; R = 0.46, *P* = 0.041) (Figures 7D–F). The relationship between gut microbiota and gut metabolites is as follows: Significant relationships between *Bacteroides_cellulosilyticus* and the metabolite kanokoside D (R = −0.49, *P* = 0.015), *Bifidobacterium_longum* and L-2,3-diaminopropanoate, 5alpha-Cholest-8-en-3beta-ol,2-Hydroxy-imipramine glucuronide (R = −0.53, *P* = 0.015; R = −0.46, *P* = 0.038; R = 0.454, *P* = 0.044); *Bifidobacterium_pseudocatenulatum* and 2-Hydroxy-imipramine glucuronide, 5alpha-Cholest-8-en-3beta-ol (R = 0.48, *P* = 0.031; R = −0.45, *P* = 0.041); *Klebsiella_oxytoca* and L-2,3-Diaminopropanoate, kanokoside D,2-Hydroxy-imipramine glucuronide (R = −0.57, *P* = 0.007; R = −0.48, *P* = 0.028; R = 0.45, *P* = 0.045); *Lactiplantibacillus_plantarum* and 2-Hydroxy-imipramine glucuronide (R = −0.49, *P* = 0.027); *Limosilactobacillus_reuteri* and 3-Methoxytyramine, androsterone (R = 0.52, *P* = 0.018; R = 0.48, *P* = 0.029); *Roseburia_inulinivorans* and niacin (nicotinic acid), l-Proline (R = 0.51, *P* = 0.021; R = 0.45, *P* = 0.041). *Rothia_dentocariosa* and 5alpha-Cholest-8-en-3beta-ol (R = 0.49, *P* = 0.026); *Ruminococcaceae_bacterium* and 2-Hydroxy-imipramine glucuronide (R = 0.61, *P* = 0.036); *Terrisporobacter_glycolicus* and niacin (Nicotinic acid), androsterone (R = −0.46, *P* = 0.038; R =-0.45, *P* = 0.042) (Figures 7G–I). The comparison of relationships between different microbial communities and different metabolites is illustrated in the Supplementary Figure 1.

### 3.8 Metagenomic library sequencing results

In this study, 20 oral, skin, and gut microbial samples were randomly selected for metagenomic sequencing in each of the patientand control groups. After removing adapters and low-quality sequences, the results were as follows: (1) For the oral samples, a total of 50.88 G of clean bases were obtained, corresponding to 50,881,122,576 clean reads after adapter removal and quality control, with an average Q2 index exceeding 98.51%; (2) For the skin samples, a total of 49.26 G of clean bases were acquired, corresponding to 49,262,012,034 clean reads after adapter removal and quality control, with an average Q2 index exceeding 98.43%; (3) For the stool samples, a total of 62.03 G of clean bases were obtained, corresponding to 62,030,892,970 clean reads after adapter removal and quality control, with an average Q2 index exceeding 98.87%. After gene prediction and redundancy removal, a non-redundant prokaryotic gene catalog was constructed, consisting of 1,929,753 genes across the oral, skin, and gut microbiomes.

#### 3.8.1 Macro gene-based observation of species diversity of flora

**Figure 8:**
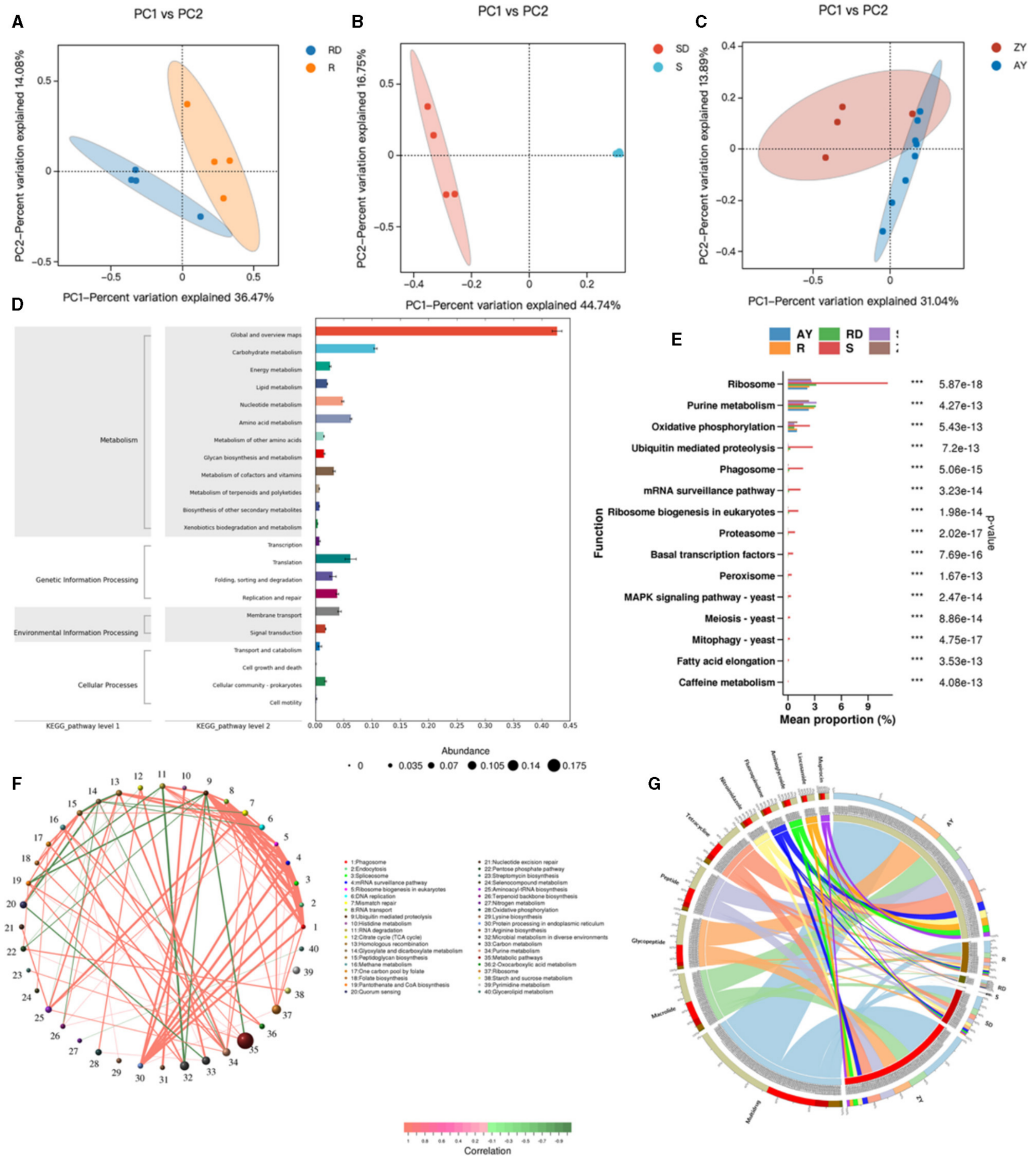
Relationship between differential microbial strains and metabolites. R, Oral samples from children with AD; S, Skin samples from children with AD; ZY, Gut samples from children with AD; RD, Oral samples from children in the healthy group; SD, Skin samples from children in the healthy group; AY, Gut samples from children in the healthy group. **(A)** PCoA map of oral strains; **(B)** PCoA map of skin strains; **(C)** PCoA map of gut strains; **(D)** KEEG map enrichment map of oral, skin, and gut flora; **(E)** KEGG_pathway_level3 abundance map of oral, skin, and gut differential flora; **(F)** Network map of differential gene correlation in the oral, skin, and gut tracts; **(G)** Antibiotic in the oral, skin, and gut tracts differential gene correlation network map.

To elucidate the overall functionality of microbial communities, we annotated functional genes of oral, skin, and gut microbiota using the eggNOG database, resulting in a total of 1,426,237 gene annotations. Based on the Bray-Curtis distance, distinct clusters were observed in the oral microbiota between the patientand control groups (Adonis test, R2 = 0.281, *P* = 0.05, Figure 8A). Similarly, in the skin microbiota, the patientand control groups formed separate clusters based on Bray-Curtis distance (Adonis test, R2 = 0.445, *P* = 0.03, Figure 8B). In the gutmicrobiota, distinct clusters were also observed between the patient and control groups based on Bray-Curtis distance (Adonis test, R2 = 0.218, *P* = 0.006, Figure 8C).

Enrichment analysis of KEGG functional pathways was conducted for the oral, skin, and gut microbiota, resulting in 430 KO pathways (Figure 8D). KEGG differential analysis demonstrated significant differences in 57 KEGG Level 3 pathways between the healthy group and the atopic AD group (Metastats analysis, *P* < 0.05) (Figure 8E). The top significant differences pathways across the oral cavity, skin, and gut were as follows: ribosome, purine metabolism, oxidative phosphorylation, ubiquitin-mediated proteolysis, Phagosome, mRNA surveillance pathway, ribosome biogenesis in eukaryotes, proteasome, basal transcription factors, peroxisome, MAPK signaling pathway, meiosis, mitophagy, fatty acid elongation, and caffeine metabolism (*P* < 0.05). Metabolism in diverse environments, citrate cycle (TCA cycle), arginine biosynthesis, glyoxylate and dicarboxylate metabolism, nitrogen metabolism, Histidine metabolism, and carbon metabolism, which exhibited significantly higher enrichment rates in the oral cavity of the patient group (*P* < 0.05). Similarly, aminoacyl-tRNA biosynthesis, carbon metabolism, mismatch repair, and ABC transporters were significantly more enriched in the oral cavity of the control group (*P* < 0.05). In the skin microbiota, compared to the control group, the patient group showed significantly higher enrichment rates in Linoleic acid metabolism and other types of O-glycan biosynthesis (*P* < 0.05), while the control group demonstrated significantly higher enrichment rates in monobactam biosynthesis, lysine biosynthesis, xylene degradation, alpha-Linolenic acid metabolism, peptidoglycan biosynthesis, phenylalanine, tyrosine, and tryptophan biosynthesis, ubiquitin-mediated proteolysis, and ABC transporters (*P* < 0.05). Additionally, acarbose and validamycin biosynthesis, polyketide sugar unit biosynthesis, various types of n-glycan biosynthesis, streptomycin biosynthesis, and styrene degradation exhibited significantly higher enrichment rates in the gut of the control group (*P* < 0.05).

Based on Level 3 KEGG pathways, we identified the top 80 most abundant functional genes in the oral cavity, skin, and gut. Using Spearman correlation analysis (including both positive and negative correlations) based on the abundance and variation of each functional gene in the samples, we performed statistical tests and selected data sets with a correlation >0.5 and a *p-*value <0.05 (Figure 8F). The network analysis revealed that the most dominant pathways in children with AD were microbial metabolism in diverse environments, carbon metabolism, purine metabolism, and metabolic pathways. Notably, metabolic pathways were negatively correlated with ubiquitin-mediated proteolysis, while microbial metabolism in diverse environments exhibited a strong negative correlation with RNA degradation.

After aligning against the Resfams database and classifying based on the mechanism of action, we identified 10 classes of antibiotic resistance genes present in the AD microbiota, with fluore quinolone, tetracycline, peptide, glycopeptide, macrolide, mupirocin, lincosamide, aminoglycoside, and nitroimidazole (Figure 8G). We identified the top 10 abundant antibiotic genes, where 3 resistance genes in the patient group exhibited significantly higher abundance than in the control group, primarily including multidrug, glycopeptide, and macrolide. Conversely, in the control group, 7 resistance genes showed significantly higher abundance than in the patient group, primarily including peptide, tetracycline, fluoroquinolone, nitroimidazole, aminoglycoside, lincosamide, and aminocoumarin.

### 3.9 The analysis of the correlation between microbial species, functional genes, and metabolites

#### 3.9.1 Correlation analysis between differential strains, metabolites, and functional genes

**Figure 9:**
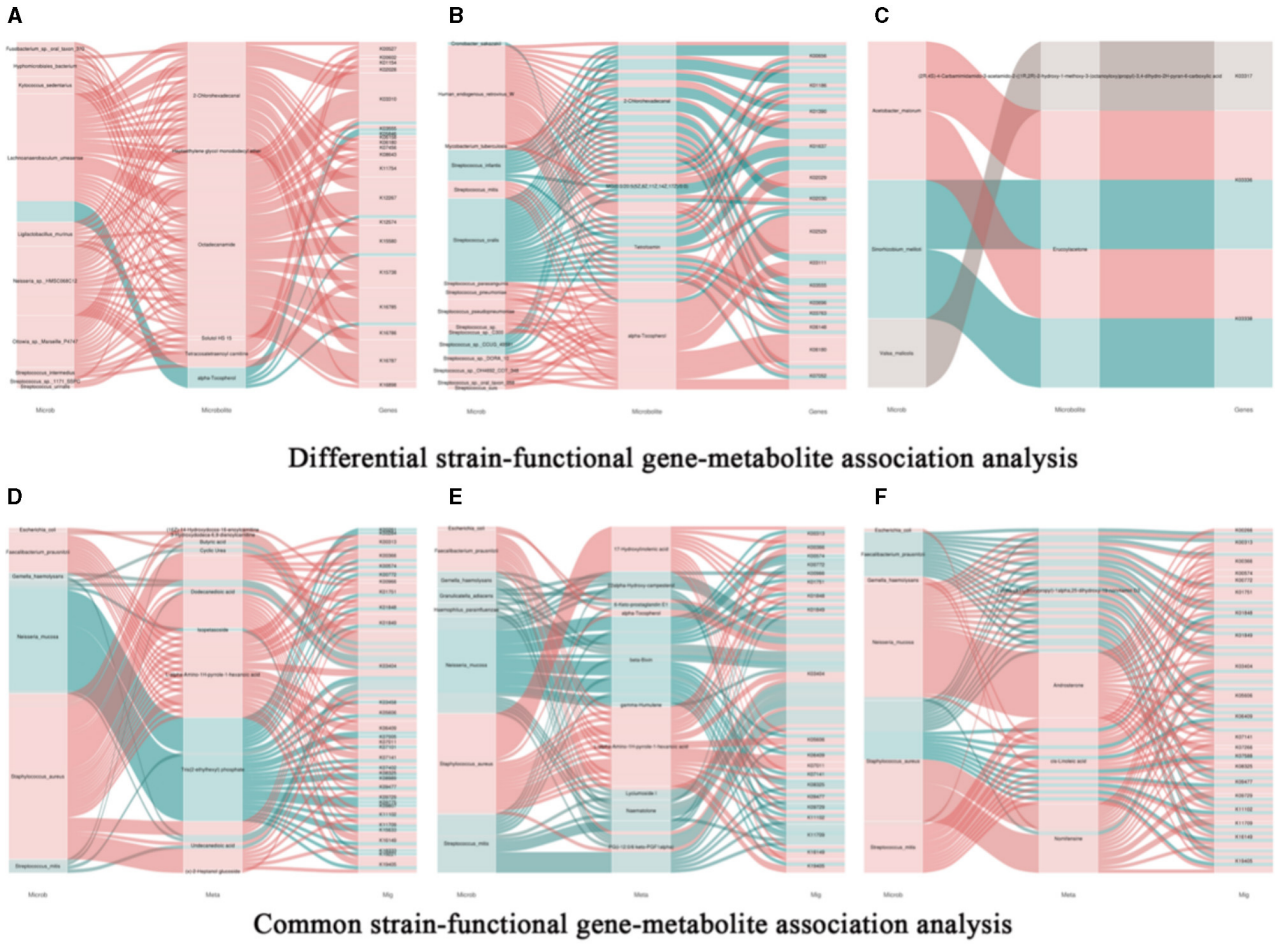
Association analysis of common bacterial species, metabolites, and functional genes in oral, skin, and guttissues of children with atopic dermatitis. **(A)** Joint analysis of oral common bacterial strains with differential metabolites and differential genes in patient and control groups, Snakey Fig. **(B)** Joint analysis of skin common bacterial strains with differential metabolites and differential genes in patient and control groups, Snakey Fig. **(C)** Joint analysis of gut common bacterial strains with differential metabolites and differential genes in patient and control groups, Snakey Fig. **(D)** Joint analysis of Oral differentiated strains with differentiated metabolites and differentiated genes joint analysis, Snakey Fig.; **(E)** Skin differentiated strains with differentiated metabolites and differentiated genes joint analysis in the patient group and the control group, Snakey Fig.; **(F)** Gut differentiated strains with differentiated metabolites and differentiated genes joint analysis in the patient group and the control group, Snakey Fig.

Based on correlation analysis, associations between oral metabolites and microbial species, as well as metabolites and functional genes, can be determined. This allows for the elucidation of multi-level regulatory relationships involving microbial species, functional genes, and metabolites. Figure 9A represents a Sankey diagram and network visualization of the oral microbiota-functional genes-metabolites relationships. The correlation threshold is set at (|r| > 0.8, *P* < 0.05). Ten microorganisms are positively correlated with 5 metabolites, including 18 pathways such as K00527. *Lachnoanaerobaculum umeaense* is negatively correlated with alpha-Tocopherol, involving pathways such as alanine or glycine: cation symporter, AGCS family (K03310), DNA mismatch repair protein MutS (K03555), osmoprotectant transport system permease protein (K05846), peptide methionine sulfoxide reductase msrA/msrB (K12267), oligopeptide transport system substrate-binding protein (K15580), and energy-coupling factor transport system permease protein (K16785). Figure 9B represents a Sankey diagram and network visualization of the skin microbiota-functional genes-metabolites relationships. The correlation threshold is set at (|r| > 0.8, *P* < 0.05). Twelve microorganisms are positively correlated with 2-chlorohexadecanal, MG [0:0/20:5(5Z, 8Z, 11Z, 14Z, 17Z)/0:0], tetrofosmin, and alpha-Tocopherol, involving 14 pathways such as formate C-acetyltransferase (K00656), sialidase-1 (K01186), IgA-specific metalloendopeptidase (K01390), and so on. Conversely, 4 microorganisms are negatively correlated with the 4 metabolites, involving 11 pathways such as K00656, K01186, K01390, K01637, K02030, K03111, K03555, K03696, K03763, K06148, and K07052. Figure 9C represents a Sankey diagram and network visualization of the gut microbiota-functional genes-metabolites relationships. The correlation threshold is set at (|r| > 0.8, *P* < 0.05). *Acetobacter_malorum* is positively correlated with the metabolite erucoylacetone, involving pathways such as 3D-(3,5/4)-trihydroxycyclohexane-1 (K03336) and 5-dehydro-2-deoxygluconokinase (K03338). *Sinorhizoblum_meliloti* is negatively correlated with the metabolite erucoylacetone, involving pathways such as K03336 and K03338 (Specific information can be found in Supplementary Table 7).

#### 3.9.2 Correlation analysis between differential strains, metabolites, and functional genes

We conducted correlation analyses between the shared microbes in the oral cavity, skin, and gut, along with their associated metabolites and metagenes. Here are our findings: (1) *E.coli* is positively correlated with cyclic urea and k03404 in the oral cavity, with 6-Keto-prostaglandin E1 and k03404 in the skin, and with nomifensine and k03404 in the gut. (2) *F. prausnitzii* is positively correlated with the oral metabolic product l-α-amino-1H-pyrrole-1-heptanoic acid and 6 genes including K00313. In the skin, it is positively correlated with l-alpha-Amino-1 H-pyrrole-1-hexanoic acid and 14 genes including K00313. In the gut, it is negatively correlated with 2alpha-(3-Hydroxypropyl)-1 alpha and 11 genes including K00366. (3) *G. haemolysans* is negatively correlated with dodecanedioic acid, undecanedioic acid, and K03404 in the oral cavity, with 6-Keto-prostaglandin E1, beta-Bixin, lyciumoside, and naematolone, and K03404 in the skin, and with cis-linoleic acid and nomifensine, and K03404 in the gut. (4) *N. mucosa* is negatively correlated with oral metabolic product tris (2-ethylhexyl) phosphate and 29 genes including K00261. In the skin, it is negatively correlated with 22alpha-Hydroxy-campesterol, K00966, K03404, K09729, K11709, beta-bixin, lyciumoside, PG (i-12:0/6 keto-PGF1alpha), naematolone, and K03404. *Neisseria mucosa* exhibits a positive correlation with the gut metabolic product 2α-(3-hydroxypropyl)-1α, 25-dihydroxy-19-norvitamin D3 and 13 genes, such as K00313. Additionally, it is positively correlated with androsterone and 17 genes, including K00266. It is negatively correlated with cis-Linoleic acid and K03404. (5) *S. aureus* is negatively correlated with (16Z)−14-Hydroxydocos-16-enoylcarnitine, K00966, 3-Hydroxydodeca-6,9-dienoylcarnitine, butyric acid, cyclic urea, dodecanedioic acid, l-alpha-amino-1H-pyrrole-1-hexanoic acid, undecanedioic acid, (x)-2-Heptanol glucoside, and K03404 in the oral cavity. In the skin, it is negatively correlated with 17-hydroxylinolenic acid, alpha-tocopherol, L-alpha-amino-1H-pyrrole-1-hexanoic acid, PG (i-12:0/6 keto-PGF1alpha), and K03404. *Staphylococcus aureus* shows a negative correlation with the gut metabolic product 2α-(3-hydroxypropyl)-1α,25-dihydroxy-19-norvitamin D3, and 11 genes, such as K00313. Additionally, it is negatively correlated with linoleic acid and the genes K01844 and K03404. (6) *S. mitis* is negatively correlated with butyric acid, dodecanedioic acid, Tris (2-ethylhexyl) phosphate, undecanedioic acid, and K03404 in the oral cavity. In the skin, it is negatively correlated with 22alpha-Hydroxy-campesterol, 6-Keto-prostaglandin E1, beta-Bixin, lyciumoside, naematolone, PG(i-12:0/6 keto-PGF1alpha), and K03404.

After filtering the gene-enriched pathways based on module connectivity, we discovered significant enrichment in the patient group in genes associated with the D-Glutamine and D-glutamate metabolism synthesis module, the cysteine and methionine metabolism module, and the glyoxylate and dicarboxylate metabolism module. In the control group, significant enrichment was observed in genes related to the conversion of carbon dioxide to acetyl-CoA in the acetyl-CoA pathway for generating energy substrates such as acetyl-CoA and pyruvate, as well as in the methylglyoxal to propionaldehyde module in the glycolysis (bacterial) pathway. Furthermore, the control group exhibited significant enrichment in genes associated with the phosphoacetyltransferase-acetate kinase pathway module, which the microbiota uses to synthesize acetate or acetates from acetyl-CoA. These findings suggest that the microbiota in children with AD have a lower potential for the synthesis of acetates and propionates (Specific information can be found in Supplementary Table 8).

## 4 Discussion

### 4.1 Dominant microbiota in different body sites of atopic dermatitis

The recent role of oral microbiota in distant site diseases has gained significant attention (Zhang et al., 2022). In our study, we observed a significant reduction in oral microbiota diversity and distinct changes in specific bacterial species in AD patients. The major bacterial species in the oral microbiota of children with AD, with relative abundance >5%, are *S. mitis (40%), N. mucosa (7.3%), G. haemolysans (7.4%), and H. parainfluenzae (5.6%)*. We found changes in the composition of the oral microbiota in AD patients that differed from oral inflammatory diseases and chronic inflammatory diseases. For example, in inflammatory bowel disease, there was an increased abundance of *Prevotella, Lactobacillus, Veillonella, and Actinomyces*. In periodontal disease, there was an increased abundance of *Fusobacterium, Prevotella, Veillonella, Campylobacter, and Actinomyces*, suggesting a potentially pro-inflammatory microenvironment in the oral cavity. However, the reduced abundance of genera in the oral cavity of AD patients compared to healthy controls suggests that the oral microbiota may have an anti-inflammatory role in AD. One of the genera with relatively high abundance, *S. mitis*, is a commensal bacterium in the human oropharynx. Currently, there are no studies suggesting a relationship between *S. mitis* and AD. However, *S. mitis* can escape the oral colonization ecosystem by expressing adhesion molecules, proteases, and toxins, leading to infective endocarditis, bacteremia, and sepsis (Mitchell, 2011). The genus *Neisseria* is one of the ten most abundant genera in the human microbiome and includes pathogenic and nonpathogenic *Neisseria* (Marri et al., 2010; Weyand, 2017). Evidence from *in vivo* studies suggests that different *Neisseria* species can colonize different parts of the nasopharynx and oral cavity, competing with other bacterial microbiota for nutrients (Donati et al., 2016). Studies have shown that non-pathogenic *Neisseria* can secrete *N. lactamica* and *N. cinerea* metabolites thereby inhibiting pathogen colonization (El et al., 2020). Thus, Neisseria mucosa may play a role in AD to suppress pathogens, but the mechanisms involved need to be further confirmed and explored.

In the skin of AD patients, the major bacterial species with relative abundance >5% include *S. aureus (14%) and S. mitis (10%)*. In this study, we found that the major bacterial species in the skin microbiota of children with AD are similar to previous literature results, with commensal streptococci and *Staphylococcus aureus* being predominant (Geoghegan et al., 2018). The pathogenesis of AD is closely related to the colonization and toxin release of *Staphylococcus aureus* (Iwamoto et al., 2019). The redistribution of filaggrin and keratin mutations in the skin stratum corneum of AD children, along with an increase in pH, creates favorable conditions for the colonization of *Staphylococcus aureus*. *Staphylococcal* enterotoxins (SEs) released by *Staphylococcus aureus* mediate the release of d-toxin and superantigens, triggering IgE-mediated mast cell degranulation, leading to an increase in Th2 cytokines and exacerbation of AD (Blicharz et al., 2022). The genus *Streptococcus* includes more than 20 pathogenic species, such as Group A *Streptococcus*, commensal *streptococci*, and *Streptococcus pneumoniae (S. pneumoniae)* (Sadowy and Hryniewicz, 2020). Although a direct relationship between the development of AD and *S. pneumoniae* was not found in previous studies (Hu et al., 2021), airway transport of pneumococci influences the dynamics of skin flora composition, initiates host immune responses, and plays an important role in childhood atopic respiratory disease (Kumpitsch et al., 2019). In addition, studies have found that gut commensal streptococci in infant AD patients are positively correlated with IgE and SCORAD indices, possibly through interactions with gut epithelial cells, which influence the progression of AD in infants through the release of inflammatory factors and reduction of short-chain fatty acids (SCFAs) (Sadowy and Hryniewicz, 2020; Kang et al., 2021). Because AD patients are often comorbid with atopic respiratory disease, *S. pneumoniae* may be a shared causative agent in AD and its comorbidities, but whether it has a bridging factor needs to be further investigated.

In the gut of AD patients, the major bacterial species with relative abundance >5% include *F. prausnit (17.2%), E. coli (10.5%), and Bacteroides_fragilis (6.4%)*. Compared to healthy individuals, AD patients exhibit a decreased diversity of gut microbiota, characterized by a significant decrease in beneficial genera such as *Lactobacillus and Bifidobacterium*, and an increase in genera such as *E. coli and Prevotella*. *IgE* susceptibility to *E. coli* antigens was found to correlate with disease severity in AD patients (Dzoro et al., 2018). This finding is consistent with previous literature results. Metagenomic analysis has shown that gut microbiota in AD patients carry additional genes associated with inflammatory responses and gut epithelial degradation.

### 4.2 Common microbiota in the three body sites of children with AD

Our analysis revealed that the oral cavity, skin, and gut of children with AD share several bacterial species: *F. prausnitzii, S. aureus, S. mitis, H. parainfluenzae, E. coli, S. salivarius, G. haemolysans, and N. mucosa*. Traceability analysis of the microbiota showed that the skin microbiota was more like the oral microbiota in children with AD compared to healthy controls, suggesting that most of the skin microbiota in AD patients may originate from the oral cavity. The inverse relationship between skin and oral metabolic pathways further suggests that there is a close link between skin and oral microbiota in AD patients. The oral cavity is the starting point of the digestive tract and is directly connected to the nasal cavity, respiratory tract, and skin. Considering the location and abundance of the oral microbiota, it is likely to be a central host for the microbiota. Chang et al. showed that the most abundant bacterial species in AD-prone populations are commensal or opportunistic pathogens in the oral cavity (Chng et al., 2016). In addition, our data show that the skin microbiota of children with AD is like the gut microbiota compared to healthy controls, and previous studies have demonstrated a high rate of transfer of *S. aureus* from parental skin to the gut microbiota of infants, suggesting that the skin microbiota may migrate to the gut in the AD environment (Lindberg et al., 2004).

The relationship between oral and gut microbiota has been explored in gastrointestinal diseases. Numerous studies have shown that the oral cavity is a source of nasal and lower respiratory tract microbiota (Atarashi et al., 2017). Even though the oral cavity and gut are environmentally isolated, more than half of the microbial species commonly detected at both sites (e.g., *Streptococcus* and *Veillonella*) show evidence of oral-intestinal translocation, even in healthy individuals (Schmidt et al., 2019). In this study, we found that *E. coli* and *N. mucosa* were present in considerable abundance in both the oral and gut tracts. This may be related to oral bacterial transmission routes. One possible route is through the bloodstream (Tsukasaki et al., 2018). Studies have shown that oral bacteria can spread to the liver and spleen in a mouse model of periodontitis, suggesting that oral inflammation plays a key role in the systemic spread of oral bacteria through the bloodstream (Pedersen et al., 2002). Another potential route is gut transmission through swallowed saliva. Ectopic colonization of the gut by oral bacteria may be associated with the pathogenesis of gastrointestinal diseases (Atarashi et al., 2017). Thus, in the genetic background of AD patients, the combined action of microbiota, including *E. coli* and *N. mucosa*, may promote Th2 dominant inflammatory responses and lead to a sustained increase in IgE levels. However, the exact mechanism requires further investigation.

### 4.3 Relationships between microbiota and metabolites in various body sites

Metabolomics provides a new tool for exploring the links between microbiota composition, host phenotype, and complex genetic traits. As metabolic regulators in the human body, the microbiota can break down complex carbohydrates, proteins, and lipids to produce a variety of metabolites that affect host metabolic homeostasis (Oliphant and Allen-Vercoe, 2019). Understanding the composition of the microbial metabolome is essential to reveal the role of the microbiota in host metabolic processes (Nowowiejska et al., 2023). Our untargeted metabolomics analysis revealed that arachidonic acid, sphingolipid signaling, Serotonergic Synapse, and fatty acid degradation pathways were significantly down-regulated in microbial metabolic pathways in children with AD. Arachidonic acid is a polyunsaturated fatty acid that is metabolized via the cyclooxygenase (COX) and lipoxygenase (LOX) pathways to produce biologically active lipid mediators, such as prostaglandins, leukotrienes, and thromboxanes, which play important roles in inflammation and immune responses (Kovács et al., 2023). Sphingolipids are components of cell membranes and are involved in cell signaling. The sphingolipid signaling pathway includes several key molecules such as ceramide, sphingosine, and sphingosine-1-phosphate (S1P), which play important roles in skin barrier function and inflammatory responses (Hannun and Obeid, 2018). Patients with AD usually have skin barrier dysfunction, and ceramides are important lipids for maintaining skin barrier function. Decreased ceramide levels correlate with the severity of atopic dermatitis, and ceramide supplementation may improve skin barrier function (Zheng et al., 2022). The 5-hydroxytryptamine signaling pathway plays a key role in emotion regulation, and psychological stress may exacerbate symptoms of AD by affecting the 5-hydroxytryptamine signaling pathway (Yang et al., 2021). Interestingly, we found that the KEGG pathway of neuroactive ligand receptors was reversed in the oral microbiota of AD children compared to the skin microbiota. This pathway is overactive in high-HRD tumors and is associated with immunosuppression in high-HRD colorectal cancer cancers (Yang et al., 2023). Therefore, the presence of high expression of neuroactive ligand receptors in the oral microbiota may play an anti-inflammatory role. In addition, sublingual immunotherapy is effective in treating atopic diseases, suggesting that the oral microbiota may generate a local anti-inflammatory microenvironment that plays an immunomodulatory role in AD skin inflammation (Roberts et al., 2018).

By analyzing the correlation between differential microbiota and metabolites, we found a close relationship between oral differential microbiota and SCFAs and lipid metabolism. Skin and gut differential microbiota were strongly associated mainly with amino acids, glycerol, and unsaturated fatty acids. It has been shown that blood components of eicosanoids such as lipoprotein A4, leukotriene B5, and docosahexaenoic acid (DHA) are significantly decreased in AD patients (Nowowiejska et al., 2023) and that dyslipidemia correlates with the severity of SCORAD score (Kim et al., 2021). Increased colonization by S. aureus was found to be associated with differences in lipid metabolism (Emmert et al., 2021). This suggests that differences in secondary products produced by lipid metabolism in AD are associated with colonization of the microbiota and points the way for subsequent studies.

### 4.4 Relationship between microbiota and genes in various body sites

We correlated the common microbiota in the oral cavity, skin, and gut with metabolites and macrogenomes. After filtering gene enrichment pathways based on modular connectivity, we observed a significant enrichment of the acetyl-CoA pathway, which produces the energy substrates acetyl-CoA and pyruvate, in controls. Some of the acetyl coenzyme A produced from the mitochondrial pathway may be transported to the cytoplasm for fatty acid synthesis contributing to lipid production in oily microbes (Strijbis and Distel, 2010). The three primary SCFAs produced by the microbiota include acetate, propionate, and butyrate, each of which has different effects on host physiology (Morrison and Preston, 2016). The pathway for acetate production is widespread in the bacterial community, with many anaerobic bacteria in the gut metabolizing and producing acetate (Thursby and Juge, 2017). In contrast, the production pathways for propionate and butyrate are more conserved and substrate-specific and are mainly produced by different bacterial subpopulations through different molecular pathways. Butyrate is produced from carbohydrates via glycolysis and the acetyl-CoA pathway, whereas propionate is formed via the succinate or propylene glycol pathway (Ríos-Covián et al., 2016; Louis and Flint, 2017). Pyruvate can be metabolized to succinate, lactate, or acetyl coenzyme A, which is then metabolized by the host to produce energy, along with SCFA such as acetate, propionate, and butyrate (Oliphant and Allen-Vercoe, 2019).

We therefore suggest that AD children may have a lower potential for synthesizing SCFAs through the three major microbiota. Studies have shown that SCFAs play an important role in regulating skin homeostasis: topical application of propionate reduces MC903-induced skin inflammation in a mouse model of AD by inhibiting IL-33 production in keratinocytes (Qiu et al., 2022); sodium butyrate ameliorates AD-induced inflammation through inhibition of the STAT1 and NF-κB pathways and may inhibit AD patients' growth of S. aureus on the skin (Traisaeng et al., 2019; Hu et al., 2023). Thus, supplementation with propionate and butyrate brings new perspectives for complementary alternative therapies for AD.

In recent years, the role of the human microbiome as a reservoir for antibiotic resistance genes (ARGs) has received increasing attention. Human and animal microbiota are recognized as hosts of antibiotic-resistant bacteria (ARB) and ARGs, and as the main mediators of ARGs transmission (Wang et al., 2020). Antibiotics can facilitate the spread of ARGs and the colonization and development of ARGs-containing bacteria in the host, thereby promoting the evolution of antibiotic-resistance genes in the microbiota (Chait et al., 2016). In this study, we characterized and compared ARGs in the oral, skin, and gut microbiota of children with AD and healthy children by macrogenomics. After categorizing ARGs according to the mechanism of action, we identified 10 classes of resistance genes commonly found in the microbiota of children with AD, mainly including fluoroquinolone, tetracycline, peptide, glycopeptide, macrolide, mupirocin, lincosamide, aminoglycoside, and nitroimidazole.

The acquisition and development of antimicrobial resistance (AMR) by commensal and pathogenic bacteria in the microbiome during childhood is largely unknown. However, studies have shown that species such as S. mitis, *S. sanginis* and *S. anginosus* in the oral microbiome have been identified as carriers of ARGs. These ARGs-carrying species may cause infectious diseases such as infective endocarditis and ventilator-associated pneumonia (Yumoto et al., 2019). According to previously published data and the present study, ARGs in the skin mainly include fluoroquinolone, β-lactamase, peptide, aminoglycoside, macrolide, and tetracycline resistance genes, which may be associated with *S. aureus*, the main pathogenic species in AD skin (Oh et al., 2016; Zhou et al., 2020). *S. aureus* on the one hand usually occupies a special position due to its relatively high virulence and ability to adapt to various environmental conditions. It is also one of the most resistant microorganisms to antimicrobial drugs and can rapidly spread clones through higher virulence and resistance to many antibiotics (Mlynarczyk-Bonikowska et al., 2022). The human gut microbiota includes a large number of ARGs, which are exchanged by horizontal gene transfer and exchanged with pathogens (Iacob and Iacob, 2019). Macrogenome sequencing provided an initial characterization of ARGs in the human gut microbiota (Bertrand et al., 2019). *E. coli* exhibits resistance to major classes of antibiotics such as β-lactams, quinolones, aminoglycosides, sulfonamides, and phosphomycins (Hesp et al., 2021).

## 5 Conclusion

This study provides the first evidence of differences in the composition of oral, skin and gut microbiota between Chinese children with AD and healthy children. By analyzing and comparing the structure and function of the skin, oral, and gut microbiota of children with AD, we found that species richness was reduced in all three sites. Combined analyses between microbiota, metabolism, and macrogenes identified key bacteria, metabolites, and pathogenic pathways that may be associated with the development of AD. This makes the microbiota and its metabolites potential biomarkers for AD, such as the common pathogenic bacteria: *S. aureus, Neisseria, S. mitis*, and *E. coli*. And in addition to the topical use of anti-inflammatory agents such as moisturizers, corticosteroids, and calcium-modulated phosphatase inhibitors; phototherapy and systemic immunosuppressive agents can be used to treat AD. Our findings also offer potential microbe-targeted therapeutic ideas for AD. For example, (1) skin barrier repair through intake of probiotics and prebiotics. Our untargeted metabolomics analysis revealed that arachidonic acid, sphingolipid signaling, 5-hydroxytryptamine signaling pathway, and fatty acid degradation pathway were significantly down-regulated in microbial metabolic pathways in children with AD. Probiotics and prebiotics can secrete a variety of metabolites and neurotransmitters (SCFAs, phenolics, 5-HT and tryptophan, etc.), which can change the intestinal mucosal permeability, and subsequently reduce the entry of harmful substances into the circulatory system to affect the skin barrier function; induce the production of anti-inflammatory cytokine IL-10 and stimulate the secretion of hypothalamic hormones, thus regulating the skin immune status (Seth et al., 2008; Levkovich et al., 2013). (2) Addition of antimicrobial peptides (AMPs): AMPs are short peptides with antimicrobial activity, which can regulate the host's natural immune system to promote pathogen clearance. Used in adult patients with moderate-to-severe AD. Omganan was effective in restoring the ecological balance of the skin, reducing *S. aureus* colonization, and increasing the microbial diversity index (Kolk et al., 2020). In this study, we jointly analyzed the antimicrobial resistance genes present in the three sites, which also provides some reference for subsequent avoidance of resistance present in the clinical use of antimicrobials. (3) Bacteriophage transplantation is a therapeutic approach to transplant functional bacteria into the skin and gastrointestinal mucosa to reconstitute microbial homeostasis and restore host function. Currently, flora transplantation methods commonly used for the treatment of AD mainly include coagulase negative staphylococci (CoNS) transplantation and fecal microbiota transplantation (FMT). They can be used to treat AD by activating the cutaneous immune system to produce antimicrobial peptides or antagonize competing invading pathogens, and by increasing the activity of the complement system to limit the colonization of pathogens with an “anti-colonizing” effect, thereby remodeling the skin and gut microbiota to restore immune homeostasis (Kim et al., 2017). In conclusion, our study provides a more comprehensive view and deeper understanding of the role of the microbiota in different parts of AD patients, and points to new directions for future diagnosis, treatment, and prognosis.

## Data Availability

The original contributions presented in the study are included in the article/Supplementary material, further inquiries can be directed to the corresponding author.
