# Supplementary material for: Changes in oral, skin, and gut microbiota in children with atopic dermatitis: a case-control study
Source: Front Microbiol. 2024 Aug 15;15:1442126. doi: 10.3389/fmicb.2024.1442126 (PMC11358084; doi:10.3389/fmicb.2024.1442126)
Supplement: Supplementary file 2 [file Data_Sheet_2.PDF]

成都中医药大学附属医院医学伦理委员会

伦理审查批件

**伦理批件号**	2022KL-059		
**项目名称**	二分二至节气下运用保和丸加减对湿疹患儿肠道菌群调控作用的临床研究		
**项目来源/申办者**	成都市科技厅		
**临床研究机构**	成都中医药大学附属医院		
**本中心主要研究者**	郭静		
**审查类别**	复审审查	**审查方式**	快速审查
**审查日期**	2022-07-21	**审查地点**	伦理委员会办公室
**审查委员**	曾彤，夏代宇		
**审查批准文件**	1. 复审申请<br>2. 临床研究方案(2022.07.10, v2.0)<br>3. 知情同意书(2022.07.10, v2.0)<br>4. 招募受试者的材料(2022.07.10, v2.0)<br>5. 研究病历和/或病例报告表，受试者日记卡和其他问卷表(2022.07.10, v2.0)<br>6. 主要研究者专业履历<br>7. 临床试验参加研究者一览表<br>8. 其他未列出的伦理审查相关材料<br>9. 科研项目立项证明		

审查意见

根据CFDA颁布实施的2020年《药物临床试验质量管理规范》、2016年《医疗器械临床试验质量管理规范》、2010年《药物临床试验伦理审查工作指导原则》，卫计委2016年颁布实施的《涉及人的生物医学研究伦理审查办法》，国家中医药管理局2010年颁布实施的《中医药临床研究伦理审查管理规范》，WMA《赫尔辛基宣言》和CIOMS《人体生物医学研究国际道德指南》等伦理原则，经本伦理委员会审查，同意按所批准的文件开展本临床研究。

1.请遵循GCP原则、按照伦理委员会批准的方案尽快启动临床研究。

2.研究过程中应特别注意：

①若发生严重不良事件以及方案规定必须报告的重要医学事件，请提交相应报告；②对临床研究方案、知情同意书、招募材料等的任何修改，及主要研究者的更换，请提交修正案审查申请表；③请按照伦理委员会规定的年度/定期跟踪审查频率，主要研究者在截止日期前1个月内提交研究进展报告；申办者应当向组长单位伦理委员会提交各个中心研究进展的汇总报告；④研究纳入了不符合纳入标准或符合排除标准的受试者，符合中止试验规定而未让受试者退出研究，给予错误治疗或剂量，给予方案禁止的合并用药等没有遵从方案开展研究的情况；或可能对受试者的权益/健康、以及研究的科学性造成不良影响等违背GCP原则的情况，请申办者或主要研究者提交违背方案报告；⑤申办者暂停/提前终止临床研究，请及时提交暂停/终止研究报告，重新启动暂停的研究，应通过"研究进展报告"申请伦理批准；⑥完成临床研究，请主要研究者提交结题报告。

3.对于涉及人类遗传资源的研究，请严格遵照人类遗传资源管理条例相关规定，及时完成向中国人类遗传资源管理办公室的申报备案工作，获得批准后才能启动研究。

**批件有效期**	2022.07.21-2025.07.20	**跟踪审查频率**	12 个月
**联系人及联系方式**	王艳桥：028-87783139，ethlcscd@126.com		
**主任委员签字**	常德贵 [signature]		

成都中医药大学附属医院医学伦理委员会

（盖章）

日期：2022年7月21日

扫描全能王 创建

成都中医药大学附属医院医学伦理委员会

伦理审查意见

伦理意见编号	2022KL-059-01		
项目名称	二分二至节气下运用保和丸加减对湿疹患儿肠道菌群调控作用的临床研究		
项目来源/申办者	成都市科技厅		
临床研究机构	成都中医药大学附属医院,		
本中心主要研究者	郭静		
审查类别	初始审查(科研项目)	审查方式	会议审查
审查日期	2022-07-01	审查地点	行政楼5楼会议室
审查委员	高天, 李明权, 曾彤, 常德贵, 谢 萍, 马喜桃, 南峰, 张朝明, 叶河江, 蒋运兰, 周建伟, 丁红, 夏代宇		
审查文件	1. 初始审查申请(科研项目)<br>2. 临床研究方案(2022.05.15, v1.0)<br>3. 知情同意书(2022.05.15, v1.0)<br>4. 招募受试者的材料(2022.05.15, v1.0)<br>5. 研究病历和/或病例报告表，受试者日记卡和其他问卷表<br>6. 主要研究者专业履历<br>7. 临床试验参加研究者一览表<br>8. 其他未列出的伦理审查相关材料<br>9. 科研项目立项证明		

审查意见

根据CFDA颁布实施的2020年《药物临床试验质量管理规范》、2016年《医疗器械临床试验质量管理规范》、2010年《药物临床试验伦理审查工作指导原则》，卫计委2016年颁布实施的《涉及人的生物医学研究伦理审查办法》，国家中医药管理局2010年颁布实施的《中医药临床研究伦理审查管理规范》，WMA《赫尔辛基宣言》和CIOMS《人体生物医学研究国际道德指南》等伦理原则，经本伦理委员会审查，意见如下：

方案部分，方案关于对照药-消风止痒颗粒的表述前后不一，请前后统一并在说明书用量范围内标注具体用法用量。补充研究团队人员参加GCP的证明资料。病例报告表不应出现患者的姓名、地址、联系电话。

知情同意书第八条未明确表述个人信息是否保密。

注：请按伦理审查意见修改或补充相关文件，如有不同观点，以复审申请的形式一并提交，请以下划线或阴影的方式标注修改内容，同时更新版本日期和版本号。

审查结论	**作必要的修正后同意**	跟踪审查频率	12 个月
主任/副主任委员签字	常德贵 [signature]		

成都中医药大学附属医院医学伦理委员会

（盖章）

日期：2022年7月6日

扫描全能王 创建
