# Supplementary material for: Changes in oral, skin, and gut microbiota in children with atopic dermatitis: a case-control study
Source: Front Microbiol. 2024 Aug 15;15:1442126. doi: 10.3389/fmicb.2024.1442126 (PMC11358084; doi:10.3389/fmicb.2024.1442126)
Supplement: Supplementary file 3 [file Data_Sheet_3.PDF]

Supplementary Table1.Analysis of differences between oral and skin microbiomes

Biomarker	Abundance	Group	LDA	Pvalue
k__Bacteria.p__Bacteroidota.c__Bacteroidia.o__Bacteroidales.f__Prevotellaceae	4.70	oral	4.01	0.028
k__Bacteria.p__Bacteroidota.c__Bacteroidia.o__Bacteroidales.f__Prevotellaceae.g__Prevotella_7	4.48	oral	4.15	0.000
k__Bacteria.p__Bacteroidota.c__Bacteroidia.o__Bacteroidales.f__Prevotellaceae.g__Prevotella_7.s__Prevotella_melaninogenica	4.41	oral	4.09	0.000
k__Bacteria.p__Firmicutes	5.80	oral	4.84	0.028
k__Bacteria.p__Firmicutes.c__Bacilli	5.76	oral	5.06	0.000
k__Bacteria.p__Firmicutes.c__Bacilli.o__Lactobacillales	5.70	oral	5.20	0.000
k__Bacteria.p__Firmicutes.c__Bacilli.o__Lactobacillales.f__Streptococcaceae	5.67	oral	5.22	0.000
k__Bacteria.p__Firmicutes.c__Bacilli.o__Lactobacillales.f__Streptococcaceae.g__Streptococcus	5.67	oral	5.22	0.000
k__Bacteria.p__Firmicutes.c__Bacilli.o__Lactobacillales.f__Streptococcaceae.g__Streptococcus.s__Streptococcus_mitis	5.61	oral	5.19	0.000
k__Bacteria.p__Firmicutes.c__Bacilli.o__Staphylococcales.f__Gemellaceae	4.90	oral	4.53	0.000
k__Bacteria.p__Firmicutes.c__Bacilli.o__Staphylococcales.f__Gemellaceae.g__Gemella	4.90	oral	4.53	0.000
k__Bacteria.p__Firmicutes.c__Bacilli.o__Staphylococcales.f__Gemellaceae.g__Gemella.s__Gemella_haemolysans	4.88	oral	4.52	0.000
k__Bacteria.p__Firmicutes.c__Negativicutes.o__Veillonellales_Selenomonadales	4.64	oral	4.14	0.047
k__Bacteria.p__Firmicutes.c__Negativicutes.o__Veillonellales_Selenomonadales.f__Veillonellaceae	4.64	oral	4.17	0.007
k__Bacteria.p__Firmicutes.c__Negativicutes.o__Veillonellales_Selenomonadales.f__Veillonellaceae.g__Veillonella	4.63	oral	4.22	0.000
k__Bacteria.p__Firmicutes.c__Negativicutes.o__Veillonellales_Selenomonadales.f__Veillonellaceae.g__Veillonella.s__Veillonella_dispar	4.53	oral	4.14	0.000
k__Bacteria.p__Fusobacteriota	4.49	oral	4.06	0.000
k__Bacteria.p__Fusobacteriota.c__Fusobacteriia	4.49	oral	4.06	0.000

k__Bacteria.p__Fusobacteriota.c__Fusobacteriia.o__Fusobacteriales	4.49	oral	4.06	0.000
k__Bacteria.p__Proteobacteria.c__Gammaproteobacteria.o__Burkholderiales.f__Neisseriaceae	5.05	oral	4.41	0.000
k__Bacteria.p__Proteobacteria.c__Gammaproteobacteria.o__Burkholderiales.f__Neisseriaceae.g__Neisseria	5.05	oral	4.51	0.000
k__Bacteria.p__Proteobacteria.c__Gammaproteobacteria.o__Burkholderiales.f__Neisseriaceae.g__Neisseria.s__Neisseria_mucosa	4.86	oral	4.31	0.000
k__Bacteria.p__Proteobacteria.c__Gammaproteobacteria.o__Enterobacterales	5.00	oral	4.02	0.045
k__Bacteria.p__Proteobacteria.c__Gammaproteobacteria.o__Enterobacterales.f__Pasteurellaceae	5.00	oral	4.52	0.000
k__Bacteria.p__Proteobacteria.c__Gammaproteobacteria.o__Enterobacterales.f__Pasteurellaceae.g__Haemophilus	4.93	oral	4.46	0.000
k__Bacteria.p__Proteobacteria.c__Gammaproteobacteria.o__Enterobacterales.f__Pasteurellaceae.g__Haemophilus.s__Haemophilus_haemolyticus	4.43	oral	4.05	0.000
k__Bacteria.p__Proteobacteria.c__Gammaproteobacteria.o__Enterobacterales.f__Pasteurellaceae.g__Haemophilus.s__Haemophilus_parainfluenzae	4.75	oral	4.25	0.000
k__Bacteria.p__Actinobacteriota	4.61	skin	4.22	0.000
k__Bacteria.p__Actinobacteriota.c__Actinobacteria	4.58	skin	4.19	0.000
k__Bacteria.p__Bacteroidota.c__Bacteroidia.o__Bacteroidales.f__Bacteroidaceae	4.41	skin	4.12	0.000
k__Bacteria.p__Bacteroidota.c__Bacteroidia.o__Bacteroidales.f__Bacteroidaceae.g__Bacteroides	4.41	skin	4.12	0.000
k__Bacteria.p__Bacteroidota.c__Bacteroidia.o__Bacteroidales.f__Prevotellaceae.g__Prevotella_9	4.39	skin	4.04	0.004
k__Bacteria.p__Bacteroidota.c__Bacteroidia.o__Bacteroidales.f__Prevotellaceae.g__Prevotella_9.s__Prevotella_copri	4.34	skin	4.00	0.004
k__Bacteria.p__Firmicutes.c__Bacilli.o__Lactobacillales.f__Lactobacillaceae	4.46	skin	4.08	0.000
k__Bacteria.p__Firmicutes.c__Bacilli.o__Staphylococcales.f__Staphylococcaceae	5.16	skin	4.84	0.000

k__Bacteria.p__Firmicutes.c__Bacilli.o__Staphylococcales.f__Staphylococcaceae.g__Staphylococcus	5.16	skin	4.84	0.000
k__Bacteria.p__Firmicutes.c__Bacilli.o__Staphylococcales.f__Staphylococcaceae.g__Staphylococcus.s__Staphylococcus_aureus	5.06	skin	4.73	0.000
k__Bacteria.p__Firmicutes.c__Clostridia	5.11	skin	4.76	0.000
k__Bacteria.p__Firmicutes.c__Clostridia.o__Lachnospirales	4.66	skin	4.32	0.000
k__Bacteria.p__Firmicutes.c__Clostridia.o__Lachnospirales.f__Lachnospiraceae	4.66	skin	4.32	0.000
k__Bacteria.p__Firmicutes.c__Clostridia.o__Oscillospirales	4.78	skin	4.44	0.000
k__Bacteria.p__Firmicutes.c__Clostridia.o__Oscillospirales.f__Ruminococcaceae	4.70	skin	4.36	0.000
k__Bacteria.p__Firmicutes.c__Clostridia.o__Oscillospirales.f__Ruminococcaceae.g__Faecalibacterium	4.56	skin	4.21	0.000
k__Bacteria.p__Firmicutes.c__Clostridia.o__Oscillospirales.f__Ruminococcaceae.g__Faecalibacterium.s__Faecalibacterium_prausnitzii	4.50	skin	4.15	0.000
k__Bacteria.p__Proteobacteria.c__Gammaproteobacteria.o__Burkholderiales	5.13	skin	4.34	0.031
k__Bacteria.p__Proteobacteria.c__Gammaproteobacteria.o__Burkholderiales.f__Burkholderiales_Incertae_Sedis	4.58	skin	4.29	0.004
k__Bacteria.p__Proteobacteria.c__Gammaproteobacteria.o__Burkholderiales.f__Burkholderiales_Incertae_Sedis.g__2013Ark19i	4.58	skin	4.29	0.004
k__Bacteria.p__Proteobacteria.c__Gammaproteobacteria.o__Burkholderiales.f__Burkholderiales_Incertae_Sedis.g__2013Ark19i.s__2013Ark19i_bacterium_2013Ark19i	4.58	skin	4.28	0.004
k__Bacteria.p__Proteobacteria.c__Gammaproteobacteria.o__Burkholderiales.f__Comamonadaceae	4.43	skin	4.12	0.000
k__Bacteria.p__Proteobacteria.c__Gammaproteobacteria.o__Burkholderiales.f__Comamonadaceae.g__Paucibacter	4.36	skin	4.05	0.000
k__Bacteria.p__Proteobacteria.c__Gammaproteobacteria.o__Burkholderiales.f__Comamonadaceae.g__Paucibacter.s__Cenchrus_americanus	4.36	skin	4.05	0.000
k__Bacteria.p__Proteobacteria.c__Gammaproteobacteria.o__Enterobacterales.f__Enterobacteri	4.70	skin	4.44	0.000

aceae				
k__Bacteria.p__Proteobacteria.c__Gammaproteobacteria.o__Enterobacterales.f__Enterobacteriaceae.g__Klebsiella	4.48	skin	4.22	0.000
k__Bacteria.p__Proteobacteria.c__Gammaproteobacteria.o__Enterobacterales.f__Enterobacteriaceae.g__Klebsiella.s__Klebsiella_pneumoniae	4.46	skin	4.20	0.000
k__Bacteria.p__Proteobacteria.c__Gammaproteobacteria.o__Pseudomonadales	4.62	skin	4.30	0.000
k__Bacteria.p__Proteobacteria.c__Gammaproteobacteria.o__Pseudomonadales.f__Moraxellaceae	4.58	skin	4.27	0.000
k__Bacteria.p__Proteobacteria.c__Gammaproteobacteria.o__Pseudomonadales.f__Moraxellaceae.g__Acinetobacter	4.40	skin	4.10	0.000

Supplementary Table2.Analysis of differences between oral and gut microbiomes

Biomarker	Abundance	Group	LDA	Pvalue
k__Bacteria.p__Bacteroidota.c__Bacteroidia	5.41	gut	4.90	0.000
k__Bacteria.p__Verrucomicrobiota.c__Verrucomicrobiae.o__Verrucomicrobiales.f__Akkermansiaceae.g__Akkermansia.s__Akkermansia_muciniphila	4.58	gut	4.30	0.000
k__Bacteria.p__Verrucomicrobiota.c__Verrucomicrobiae.o__Verrucomicrobiales.f__Akkermansiaceae.g__Akkermansia	4.58	gut	4.30	0.000
k__Bacteria.p__Bacteroidota.c__Bacteroidia.o__Bacteroidales.f__Bacteroidaceae.g__Bacteroides.s__Bacteroides_vulgatus	4.41	gut	4.10	0.000
k__Bacteria.p__Bacteroidota.c__Bacteroidia.o__Bacteroidales.f__Bacteroidaceae.g__Bacteroides	5.29	gut	4.99	0.000
k__Bacteria.p__Verrucomicrobiota	4.58	gut	4.30	0.000
k__Bacteria.p__Firmicutes.c__Clostridia.o__Oscillospirales.f__Ruminococcaceae.g__Subdoligranulum	4.59	gut	4.31	0.000
k__Bacteria.p__Proteobacteria.c__Gammaproteobacteria.o__Enterobacterales.f__Enterobacteriaceae.g__Escherichia_Shigella.s__Escherichia_coli	5.01	gut	4.69	0.000
k__Bacteria.p__Verrucomicrobiota.c__Verrucomicrobiae.o__Verrucomicrobiales	4.58	gut	4.30	0.000

k__Bacteria.p__Firmicutes.c__Clostridia.o__Lachnospirales.f__Lachnospiraceae.g__Lachnospiraceae_NK4A136_group	4.29	gut	4.02	0.000
k__Bacteria.p__Actinobacteriota.c__Actinobacteria.o__Bifidobacteriales	4.43	gut	4.16	0.000
k__Bacteria.p__Firmicutes.c__Clostridia.o__Lachnospirales.f__Lachnospiraceae	5.05	gut	4.76	0.000
k__Bacteria.p__Proteobacteria.c__Gammaproteobacteria.o__Enterobacterales.f__Enterobacteriaceae.g__Escherichia_Shigella	5.01	gut	4.69	0.000
k__Bacteria.p__Firmicutes.c__Clostridia.o__Oscillospirales.f__Ruminococcaceae.g__Faecalibacterium	5.16	gut	4.84	0.000
k__Bacteria.p__Firmicutes.c__Negativicutes.o__Veillonellales_Selenomonadales.f__Selenomonadaceae.g__Megamonas	4.55	gut	4.24	0.000
k__Bacteria.p__Bacteroidota.c__Bacteroidia.o__Bacteroidales.f__Bacteroidaceae.g__Bacteroides.s__Phocaeicola_vulgatus	4.38	gut	4.10	0.000
k__Bacteria.p__Firmicutes.c__Clostridia	5.58	gut	5.27	0.000
k__Bacteria.p__Verrucomicrobiota.c__Verrucomicrobiae.o__Verrucomicrobiales.f__Akkermansiaceae	4.58	gut	4.30	0.000
k__Bacteria.p__Actinobacteriota.c__Actinobacteria.o__Bifidobacteriales.f__Bifidobacteriaceae	4.43	gut	4.16	0.000
k__Bacteria.p__Firmicutes.c__Clostridia.o__Oscillospirales.f__Ruminococcaceae.g__Subdoligranulum.s__Faecalibacterium_prausnitzii	4.56	gut	4.28	0.000
k__Bacteria.p__Firmicutes.c__Clostridia.o__Oscillospirales	5.36	gut	5.05	0.000
k__Bacteria.p__Firmicutes.c__Clostridia.o__Lachnospirales	5.05	gut	4.76	0.000
k__Bacteria.p__Bacteroidota	5.41	gut	4.90	0.000
k__Bacteria.p__Proteobacteria.c__Gammaproteobacteria.o__Enterobacterales.f__Enterobacteriaceae	5.09	gut	4.77	0.000
k__Bacteria.p__Firmicutes.c__Negativicutes.o__Veillonellales_Selenomonadales.f__Selenomonadaceae.g__Megamonas.s__Megamonas_funiformis	4.55	gut	4.22	0.000
k__Bacteria.p__Firmicutes.c__Clostridia.o__Lachnospirales.f__Lachnospiraceae.g__Lachnospiraceae_NK4A136_group.s__unclassified_Lachnospiraceae_NK4A136_group	4.27	gut	4.01	0.000

k__Bacteria.p__Bacteroidota.c__Bacteroidia.o__Bacteroidales.f__Bacteroidaceae	5.29	gut	4.99	0.000
k__Bacteria.p__Firmicutes.c__Clostridia.o__Oscillospirales.f__Ruminococcaceae	5.33	gut	5.02	0.000
k__Bacteria.p__Actinobacteriota.c__Actinobacteria.o__Bifidobacteriales.f__Bifidobacteriaceae.g__Bifidobacterium	4.43	gut	4.17	0.000
k__Bacteria.p__Firmicutes.c__Clostridia.o__Lachnospirales.f__Lachnospiraceae.g__Blautia	4.24	gut	4.03	0.000
k__Bacteria.p__Bacteroidota.c__Bacteroidia.o__Bacteroidales.f__Bacteroidaceae.g__Bacteroides.s__Bacteroides_fragilis	4.84	gut	4.52	0.000
k__Bacteria.p__Firmicutes.c__Clostridia.o__Oscillospirales.f__Ruminococcaceae.g__Faecalibacterium.s__Faecalibacterium_prausnitzii	5.13	gut	4.81	0.000
k__Bacteria.p__Actinobacteriota.c__Actinobacteria.o__Bifidobacteriales.f__Bifidobacteriaceae.g__Bifidobacterium.s__Bifidobacterium_pseudocatenulatum	4.39	gut	4.12	0.000
k__Bacteria.p__Bacteroidota.c__Bacteroidia.o__Bacteroidales	5.41	gut	4.95	0.000
k__Bacteria.p__Verrucomicrobiota.c__Verrucomicrobiae	4.58	gut	4.30	0.000
k__Bacteria.p__Proteobacteria.c__Gammaproteobacteria.o__Burkholderiales.f__Neisseriaceae.g__Neisseria.s__Neisseria_mucosa	4.86	oral	4.57	0.000
k__Bacteria.p__Proteobacteria.c__Gammaproteobacteria.o__Burkholderiales.f__Neisseriaceae.g__Neisseria.s__Neisseria_flavescens	4.37	oral	4.11	0.000
k__Bacteria.p__Bacteroidota.c__Bacteroidia.o__Bacteroidales.f__Prevotellaceae.g__Prevotella_7.s__Prevotella_melaninogenica	4.41	oral	4.13	0.000
k__Bacteria.p__Firmicutes.c__Bacilli	5.76	oral	5.42	0.000
k__Bacteria.p__Firmicutes.c__Negativicutes.o__Veillonellales_Selenomonadales.f__Veillonellaceae.g__Veillonella	4.63	oral	4.25	0.000
k__Bacteria.p__Proteobacteria.c__Gammaproteobacteria	5.35	oral	4.51	0.002
k__Bacteria.p__Firmicutes.c__Bacilli.o__Lactobacillales.f__Carnobacteriaceae	4.48	oral	4.15	0.000
k__Bacteria.p__Bacteroidota.c__Bacteroidia.o__Bacteroidales.f__Prevotellaceae.g__Prevotella_7	4.48	oral	4.20	0.000
k__Bacteria.p__Firmicutes.c__Bacilli.o__Staph	4.90	oral	4.58	0.000

ylococcales.f__Gemellaceae.g__Gemella				
k__Bacteria.p__Bacteroidota.c__Bacteroidia.o__Bacteroidales.f__Prevotellaceae	4.70	oral	4.08	0.000
k__Bacteria.p__Firmicutes.c__Bacilli.o__Staphylococcales.f__Gemellaceae	4.90	oral	4.58	0.000
k__Bacteria.p__Firmicutes.c__Bacilli.o__Staphylococcales.f__Gemellaceae.g__Gemella.s__Gemella_haemolysans	4.88	oral	4.56	0.000
k__Bacteria.p__Proteobacteria.c__Gammaproteobacteria.o__Enterobacterales.f__Pasteurellaceae	5.00	oral	4.57	0.000
k__Bacteria.p__Firmicutes.c__Bacilli.o__Lactobacillales.f__Streptococcaceae.g__Streptococcus	5.67	oral	5.34	0.000
k__Bacteria.p__Proteobacteria.c__Gammaproteobacteria.o__Burkholderiales.f__Neisseriaceae.g__Neisseria	5.05	oral	4.76	0.000
k__Bacteria.p__Proteobacteria	5.35	oral	4.51	0.002
k__Bacteria.p__Firmicutes.c__Bacilli.o__Lactobacillales.f__Carnobacteriaceae.g__Granulicatella	4.48	oral	4.15	0.000
k__Bacteria.p__Proteobacteria.c__Gammaproteobacteria.o__Enterobacterales.f__Pasteurellaceae.g__Haemophilus.s__Haemophilus_parainfluenzae	4.75	oral	4.23	0.000
k__Bacteria.p__Proteobacteria.c__Gammaproteobacteria.o__Burkholderiales.f__Neisseriaceae	5.05	oral	4.76	0.000
k__Bacteria.p__Firmicutes.c__Negativicutes.o__Veillonellales_Selenomonadales.f__Veillonellaceae.g__Veillonella.s__Veillonella_dispar	4.53	oral	4.17	0.000
k__Bacteria.p__Firmicutes.c__Bacilli.o__Lactobacillales.f__Streptococcaceae.g__Streptococcus.s__Streptococcus_mitis	5.61	oral	5.31	0.000
k__Bacteria.p__Firmicutes.c__Bacilli.o__Lactobacillales.f__Streptococcaceae	5.67	oral	5.34	0.000
k__Bacteria.p__Proteobacteria.c__Gammaproteobacteria.o__Enterobacterales.f__Pasteurellaceae.g__Haemophilus.s__Haemophilus_haemolyticus	4.43	oral	4.09	0.000
k__Bacteria.p__Bacteroidota.c__Bacteroidia.o__Bacteroidales.f__Porphyromonadaceae.g__Porphyromonas	4.35	oral	4.07	0.000
k__Bacteria.p__Firmicutes	5.80	oral	4.82	0.020
k__Bacteria.p__Firmicutes.c__Bacilli.o__Staph	4.90	oral	4.58	0.000

ylococcales				
k__Bacteria.p__Proteobacteria.c__Gammaproteobacteria.o__Burkholderiales	5.08	oral	4.77	0.000
k__Bacteria.p__Bacteroidota.c__Bacteroidia.o__Bacteroidales.f__Porphyromonadaceae	4.35	oral	4.07	0.000
k__Bacteria.p__Proteobacteria.c__Gammaproteobacteria.o__Enterobacterales.f__Pasteurellaceae.g__Haemophilus	4.93	oral	4.48	0.000
k__Bacteria.p__Bacteroidota.c__Bacteroidia.o__Flavobacteriales	4.37	oral	4.04	0.000
k__Bacteria.p__Firmicutes.c__Bacilli.o__Lactobacillales	5.70	oral	5.37	0.000

Supplementary Table3.Analysis of differences between gut and skin microbiomes

Biomarker	Abundance	Group	LDA	Pvalue
k__Bacteria.p__Proteobacteria.c__Gammaproteobacteria.o__Enterobacterales.f__Enterobacteriaceae.g__Escherichia_Shigella.s__Escherichia_coli	5.01	gut	4.59	0.005
k__Bacteria.p__Proteobacteria.c__Gammaproteobacteria.o__Enterobacterales.f__Enterobacteriaceae.g__Escherichia_Shigella	5.01	gut	4.59	0.005
k__Bacteria.p__Firmicutes.c__Negativicutes.o__Veillonellales_Selenomonadales.f__Selenomonadaceae	4.55	gut	4.27	0.036
k__Bacteria.p__Firmicutes.c__Negativicutes	4.83	gut	4.41	0.031
k__Bacteria.p__Firmicutes.c__Clostridia.o__Oscillospirales.f__Ruminococcaceae.g__Subdoligranulum	4.59	gut	4.17	0.005
k__Bacteria.p__Firmicutes.c__Clostridia.o__Oscillospirales.f__Ruminococcaceae.g__Faecalibacterium.s__Faecalibacterium_prausnitzii	5.13	gut	4.76	0.000
k__Bacteria.p__Firmicutes.c__Clostridia.o__Oscillospirales.f__Ruminococcaceae.g__Faecalibacterium	5.16	gut	4.78	0.000

k__Bacteria.p__Firmicutes.c__Clostridia.o__ Oscillospirales.f__Ruminococcaceae	5.33	gut	4.94	0.000
k__Bacteria.p__Firmicutes.c__Clostridia.o__ Oscillospirales	5.36	gut	4.96	0.000
k__Bacteria.p__Firmicutes.c__Clostridia.o__ Lachnospirales.f__Lachnospiraceae	5.05	gut	4.51	0.000
k__Bacteria.p__Firmicutes.c__Clostridia.o__ Lachnospirales	5.05	gut	4.51	0.000
k__Bacteria.p__Firmicutes.c__Clostridia	5.58	gut	5.11	0.000
k__Bacteria.p__Bacteroidota.c__Bacteroidia. o__Bacteroidales.f__Prevotellaceae	4.66	gut	4.10	0.000
k__Bacteria.p__Bacteroidota.c__Bacteroidia. o__Bacteroidales.f__Bacteroidaceae.g__Bacte roides.s__Phocaeicola_vulgatus	4.38	gut	4.10	0.034
k__Bacteria.p__Bacteroidota.c__Bacteroidia. o__Bacteroidales.f__Bacteroidaceae.g__Bacte roides.s__Bacteroides_vulgatus	4.41	gut	4.08	0.010
k__Bacteria.p__Bacteroidota.c__Bacteroidia. o__Bacteroidales.f__Bacteroidaceae.g__Bacte roides.s__Bacteroides_fragilis	4.84	gut	4.48	0.000
k__Bacteria.p__Bacteroidota.c__Bacteroidia. o__Bacteroidales.f__Bacteroidaceae.g__Bacte roides	5.29	gut	4.93	0.000
k__Bacteria.p__Bacteroidota.c__Bacteroidia. o__Bacteroidales.f__Bacteroidaceae	5.29	gut	4.93	0.000
k__Bacteria.p__Bacteroidota.c__Bacteroidia. o__Bacteroidales	5.41	gut	4.98	0.000
k__Bacteria.p__Bacteroidota.c__Bacteroidia	5.41	gut	4.93	0.000
k__Bacteria.p__Bacteroidota	5.41	gut	4.93	0.000
k__Bacteria.p__Proteobacteria.c__Gammapro teobacteria.o__Pseudomonadales.f__Moraxell aceae	4.58	skin	4.11	0.000
k__Bacteria.p__Proteobacteria.c__Gammapro teobacteria.o__Pseudomonadales	4.62	skin	4.15	0.000
k__Bacteria.p__Proteobacteria.c__Gammapro teobacteria.o__Enterobacterales.f__Pasteurell aceae	4.52	skin	4.01	0.001
k__Bacteria.p__Proteobacteria.c__Gammapro teobacteria.o__Burkholderiales.f__Neisseriac eae.g__unclassified_Neisseriaceae	4.32	skin	4.01	0.001
k__Bacteria.p__Proteobacteria.c__Gammapro teobacteria.o__Burkholderiales.f__Neisseriac eae.g__Neisseria.s__Neisseria_mucosa	4.47	skin	4.14	0.000
k__Bacteria.p__Proteobacteria.c__Gammapro	4.59	skin	4.28	0.000

teobacteria.o__Burkholderiales.f__Neisseriaceae.g__Neisseria				
k__Bacteria.p__Proteobacteria.c__Gammaproteobacteria.o__Burkholderiales.f__Neisseriaceae	4.78	skin	4.46	0.000
k__Bacteria.p__Proteobacteria.c__Gammaproteobacteria.o__Burkholderiales.f__Comamonadaceae	4.43	skin	4.04	0.000
k__Bacteria.p__Proteobacteria.c__Gammaproteobacteria.o__Burkholderiales.f__Burkholderiales_Incertae_Sedis.g__2013Ark19i.s__2013Ark19i_bacterium_2013Ark19i	4.58	skin	4.35	0.013
k__Bacteria.p__Proteobacteria.c__Gammaproteobacteria.o__Burkholderiales.f__Burkholderiales_Incertae_Sedis.g__2013Ark19i	4.58	skin	4.35	0.013
k__Bacteria.p__Proteobacteria.c__Gammaproteobacteria.o__Burkholderiales.f__Burkholderiales_Incertae_Sedis	4.58	skin	4.32	0.013
k__Bacteria.p__Proteobacteria.c__Gammaproteobacteria.o__Burkholderiales	5.13	skin	4.81	0.000
k__Bacteria.p__Proteobacteria.c__Gammaproteobacteria	5.46	skin	4.83	0.004
k__Bacteria.p__Proteobacteria	5.48	skin	4.87	0.002
k__Bacteria.p__Firmicutes.c__Bacilli.o__Staphylococcales.f__Staphylococcaceae.g__Staphylococcus.s__Staphylococcus_aureus	5.06	skin	4.76	0.000
k__Bacteria.p__Firmicutes.c__Bacilli.o__Staphylococcales.f__Staphylococcaceae.g__Staphylococcus	5.16	skin	4.87	0.000
k__Bacteria.p__Firmicutes.c__Bacilli.o__Staphylococcales.f__Staphylococcaceae	5.16	skin	4.87	0.000
k__Bacteria.p__Firmicutes.c__Bacilli.o__Staphylococcales	5.19	skin	4.90	0.000
k__Bacteria.p__Firmicutes.c__Bacilli.o__Lactobacillales.f__Streptococcaceae.g__Streptococcus.s__Streptococcus_mitis	4.94	skin	4.64	0.000
k__Bacteria.p__Firmicutes.c__Bacilli.o__Lactobacillales.f__Streptococcaceae.g__Streptococcus	5.09	skin	4.68	0.000
k__Bacteria.p__Firmicutes.c__Bacilli.o__Lactobacillales.f__Streptococcaceae	5.09	skin	4.69	0.000
k__Bacteria.p__Firmicutes.c__Bacilli.o__Lactobacillales.f__Lactobacillaceae	4.46	skin	4.07	0.000
k__Bacteria.p__Firmicutes.c__Bacilli.o__Lac	5.26	skin	4.87	0.000

tobacillales				
k__Bacteria.p__Campylobacterota.c__Campylobacteria.o__Campylobacterales	4.43	skin	4.18	0.000
k__Bacteria.p__Campylobacterota.c__Campylobacteria	4.43	skin	4.18	0.000
k__Bacteria.p__Campylobacterota	4.43	skin	4.18	0.000
k__Bacteria.p__Actinobacteriota.c__Actinobacteria	4.58	skin	4.03	0.000
k__Bacteria.p__Actinobacteriota	4.61	skin	4.05	0.000
