# Supplementary material for: Changes in oral, skin, and gut microbiota in children with atopic dermatitis: a case-control study
Source: Front Microbiol. 2024 Aug 15;15:1442126. doi: 10.3389/fmicb.2024.1442126 (PMC11358084; doi:10.3389/fmicb.2024.1442126)
Supplement: Supplementary file 4 [file Data_Sheet_4.PDF]

Supplementary Table 4.Similarity analysis of oral and skin strains of bacteria.

taxonomy	Oral (abundances)	Skin (abundances)
k__Bacteria; p__Firmicutes; c__Bacilli; o__Lactobacillales; f__Streptococcaceae; g__Streptococcus; s__Streptococcus_mitis;	148229	19443
k__Bacteria; p__Firmicutes; c__Bacilli; o__Staphylococcales; f__Gemellaceae; g__Gemella; s__Gemella_haemolysans;	27414	2133
k__Bacteria; p__Proteobacteria; c__Gammaproteobacteria; o__Burkholderiales; f__Neisseriaceae; g__Neisseria; s__Neisseria_mucosa;	26759	6659
k__Bacteria; p__Proteobacteria; c__Gammaproteobacteria; o__Enterobacterales; f__Pasteurellaceae; g__Haemophilus; s__Haemophilus_parainfluenzae;	20740	3953
k__Bacteria; p__Firmicutes; c__Negativicutes; o__Veillonellales_Selenomonadales; f__Veillonellaceae; g__Veillonella; s__Veillonella_dispar;	12162	1054
k__Bacteria; p__Proteobacteria; c__Gammaproteobacteria; o__Enterobacterales; f__Pasteurellaceae; g__Haemophilus; s__Haemophilus_haemolyticus;	10196	1413
k__Bacteria; p__Bacteroidota; c__Bacteroidia; o__Bacteroidales; f__Prevotellaceae; g__Prevotella_7; s__Prevotella_melaninogenica;	8902	233
k__Bacteria; p__Proteobacteria; c__Gammaproteobacteria; o__Burkholderiales; f__Neisseriaceae; g__Neisseria; s__Neisseria_flavescens;	8134	923
k__Bacteria; p__Firmicutes; c__Bacilli; o__Lactobacillales; f__Streptococcaceae; g__Streptococcus; s__Streptococcus_sanguinis;	5869	1645
k__Bacteria; p__Firmicutes; c__Bacilli; o__Lactobacillales; f__Carnobacteriaceae; g__Granulicatella; s__Granulicatella_adiacens;	5824	1470
k__Bacteria; p__Bacteroidota; c__Bacteroidia; o__Flavobacteriales; f__Weeksellaceae; g__Bergeyella; s__unclassified_Bergeyella;	5657	825
k__Bacteria; p__Firmicutes; c__Bacilli; o__Lactobacillales; f__Carnobacteriaceae; g__Granulicatella; s__Granulicatella_elegans;	5304	1142
k__Bacteria; p__Firmicutes; c__Bacilli; o__Lactobacillales; f__Streptococcaceae; g__Streptococcus; s__Streptococcus_pneumoniae;	4906	351
k__Bacteria; p__Bacteroidota; c__Bacteroidia; o__Bacteroidales; f__Prevotellaceae; g__Alloprevotella; s__Prevotellaceae_bacterium_Marseille_P2826;	4246	877
k__Bacteria; p__Bacteroidota; c__Bacteroidia; o__Bacteroidales; f__Porphyromonadaceae; g__Porphyromonas; s__unclassified_Porphyromonas;	3944	305

k__Bacteria; p__Bacteroidota; c__Bacteroidia; o__Bacteroidales; f__Porphyromonadaceae; g__Porphyromonas; s__Porphyromonas_pasteri;	3657	483
k__Bacteria; p__Proteobacteria; c__Gammaproteobacteria; o__Enterobacterales; f__Pasteurellaceae; g__Actinobacillus; s__Haemophilus_parahaemolyticus;	2954	838
k__Bacteria; p__Proteobacteria; c__Gammaproteobacteria; o__Burkholderiales; f__Neisseriaceae; g__Neisseria; s__Neisseria_cinerea;	2611	624
k__Bacteria; p__Fusobacteriota; c__Fusobacteriia; o__Fusobacteriales; f__Leptotrichiaceae; g__Streptobacillus; s__unclassified_Streptobacillus;	2411	304
k__Bacteria; p__Firmicutes; c__Bacilli; o__Lactobacillales; f__Streptococcaceae; g__Streptococcus; s__Streptococcus_salivarius;	2235	1666
k__Bacteria; p__Firmicutes; c__Bacilli; o__Lactobacillales; f__Aerococcaceae; g__Abiotrophia; s__Abiotrophia_defectiva;	2234	964
k__Bacteria; p__Fusobacteriota; c__Fusobacteriia; o__Fusobacteriales; f__Fusobacteriaceae; g__Fusobacterium; s__Fusobacterium_pseudoperiodonticum;	2194	142
k__Bacteria; p__Proteobacteria; c__Gammaproteobacteria; o__Burkholderiales; f__Burkholderiaceae; g__Lautropia; s__Lautropia_mirabilis;	2083	621
k__Bacteria; p__Firmicutes; c__Bacilli; o__Lactobacillales; f__Streptococcaceae; g__Streptococcus; s__Streptococcus_genomosp._C4;	2027	343
k__Bacteria; p__Actinobacteriota; c__Actinobacteria; o__Micrococcales; f__Micrococcaceae; g__Rothia; s__Rothia_dentocariosa;	1978	739
k__Bacteria; p__Firmicutes; c__Bacilli; o__Lactobacillales; f__Streptococcaceae; g__Streptococcus; s__Streptococcus_oralis;	1956	516
k__Bacteria; p__Firmicutes; c__Negativicutes; o__Veillonellales_Selenomonadales; f__Veillonellaceae; g__Veillonella; s__Veillonella_parvula;	1650	301
k__Bacteria; p__Fusobacteriota; c__Fusobacteriia; o__Fusobacteriales; f__Fusobacteriaceae; g__Fusobacterium; s__Fusobacterium_nucleatum;	1405	411
k__Bacteria; p__Firmicutes; c__Negativicutes; o__Veillonellales_Selenomonadales; f__Veillonellaceae; g__Veillonella; s__Veillonella_massiliensis;	1360	480
k__Bacteria; p__Patescibacteria; c__Saccharimonadia; o__Saccharimonadales; f__uncultured_Candidatus_Saccharibacteria_bacterium; g__uncultured_Candidatus_Saccharibacteria_bacterium;	1304	202

s__uncultured_Candidatus_Saccharibacteria_bacterium;		
k__Bacteria; p__Proteobacteria; c__Gammaproteobacteria; o__Enterobacterales; f__Pasteurellaceae; g__Aggregatibacter; s__Aggregatibacter_segnis;	1274	302
k__Bacteria; p__Fusobacteriota; c__Fusobacteriia; o__Fusobacteriales; f__Leptotrichiaceae; g__Leptotrichia; s__unclassified_Leptotrichia;	1142	189
k__Bacteria; p__Firmicutes; c__Bacilli; o__Lactobacillales; f__Streptococcaceae; g__Streptococcus; s__Streptococcus_cristatus;	1056	309
k__Bacteria; p__Firmicutes; c__Bacilli; o__Lactobacillales; f__Streptococcaceae; g__Streptococcus; s__Streptococcus_infantis;	980	314
k__Bacteria; p__Bacteroidota; c__Bacteroidia; o__Flavobacteriales; f__Flavobacteriaceae; g__Capnocytophaga; s__Capnocytophaga_granulosa;	977	167
k__Bacteria; p__Firmicutes; c__Bacilli; o__Lactobacillales; f__Streptococcaceae; g__Streptococcus; s__Streptococcus_parasanguinis;	747	529
k__Bacteria; p__Proteobacteria; c__Gammaproteobacteria; o__Enterobacterales; f__Pasteurellaceae; g__Actinobacillus; s__Haemophilus_paraphrohaemolyticus_HK411;	694	142
k__Bacteria; p__Firmicutes; c__Bacilli; o__Staphylococcales; f__Gemellaceae; g__Gemella; s__Gemella_sanguinis;	624	124
k__Bacteria; p__Bacteroidota; c__Bacteroidia; o__Flavobacteriales; f__Flavobacteriaceae; g__Capnocytophaga; s__Capnocytophaga_sputigena;	590	110
k__Bacteria; p__Campylobacterota; c__Campylobacteria; o__Campylobacterales; f__Campylobacteraceae; g__Campylobacter; s__Campylobacter_concisus;	520	114
k__Bacteria; p__Bacteroidota; c__Bacteroidia; o__Flavobacteriales; f__Flavobacteriaceae; g__Capnocytophaga; s__Capnocytophaga_gingivalis;	496	387
k__Bacteria; p__Proteobacteria; c__Gammaproteobacteria; o__Burkholderiales; f__Neisseriaceae; g__Neisseria; s__Neisseria_subflava;	495	146
k__Bacteria; p__Proteobacteria; c__Gammaproteobacteria; o__Burkholderiales; f__Neisseriaceae; g__Neisseria; s__Neisseria_oralis;	494	287
k__Bacteria; p__Actinobacteriota; c__Actinobacteria; o__Micrococcales; f__Micrococcaceae; g__Rothia; s__Rothia_aeria;	469	212
k__Bacteria; p__Firmicutes; c__Bacilli; o__Staphylococcales; f__Gemellaceae; g__Gemella; s__Gemella_morbillorum;	452	142
k__Bacteria; p__Proteobacteria; c__Gammaproteobacteria; o__Enterobacterales; f__Pasteurellaceae; g__Haemophilus; s__Haemophilus_sputorum;	446	170

k__Bacteria; p__Proteobacteria; c__Gammaproteobacteria; o__Enterobacterales; f__Pasteurellaceae; g__Aggregatibacter; s__Aggregatibacter_aphrophilus;	389	122
k__Bacteria; p__Firmicutes; c__Bacilli; o__Lactobacillales; f__Streptococcaceae; g__Streptococcus; s__Streptococcus_gordonii;	377	237
k__Bacteria; p__Proteobacteria; c__Gammaproteobacteria; o__Pseudomonadales; f__Moraxellaceae; g__Moraxella; s__Moraxella_sp.;	374	224
k__Bacteria; p__Fusobacteriota; c__Fusobacteriia; o__Fusobacteriales; f__Leptotrichiaceae; g__Leptotrichia; s__Leptotrichia_sp._oral_taxon_212;	351	111
k__Bacteria; p__Proteobacteria; c__Gammaproteobacteria; o__Burkholderiales; f__Burkholderiales_Incertae_Sedis; g__2013Ark19i; s__2013Ark19i_bacterium_2013Ark19i;	325	5338
k__Bacteria; p__Proteobacteria; c__Gammaproteobacteria; o__Burkholderiales; f__Neisseriaceae; g__Neisseria; s__Neisseria_meningitidis;	254	111
k__Bacteria; p__Proteobacteria; c__Gammaproteobacteria; o__Burkholderiales; f__Comamonadaceae; g__Paucibacter; s__Cenchrus_americanus;	165	5547
k__Bacteria; p__Firmicutes; c__Bacilli; o__Lactobacillales; f__Streptococcaceae; g__Streptococcus; s__Streptococcus_thermophilus;	161	120
k__Bacteria; p__Proteobacteria; c__Gammaproteobacteria; o__Enterobacterales; f__Pasteurellaceae; g__Haemophilus; s__Haemophilus_influenzae;	158	105
k__Bacteria; p__Firmicutes; c__Clostridia; o__Clostridia_UCG_014; f__unclassified_Clostridia_UCG_014; g__unclassified_Clostridia_UCG_014; s__unclassified_Clostridia_UCG_014;	148	100
k__Bacteria; p__Campylobacterota; c__Campylobacteria; o__Campylobacterales; f__Helicobacteraceae; g__Helicobacter; s__Helicobacter_japonicus;	123	4514
k__Bacteria; p__Firmicutes; c__Negativicutes; o__Veillonellales_Selenomonadales; f__Veillonellaceae; g__Dialister; s__Dialister_invisus;	105	229

Supplementary Table 5.Similarity analysis of oral and gut strains of bacteria.

taxonomy	Oral (abundances)	Gut (abundances)
k__Bacteria; p__Proteobacteria; c__Gammaproteobacteria; o__Enterobacterales; f__Enterobacteriaceae; g__Escherichia_Shigella; s__Escherichia_coli;	21	23987

k__Bacteria; p__Verrucomicrobiota; c__Verrucomicrobiae; o__Verrucomicrobiales; f__Akkermansiaceae; g__Akkermansia; s__Akkermansia_muciniphila;	25	8810
k__Bacteria; p__Firmicutes; c__Bacilli; o__Lactobacillales; f__Streptococcaceae; g__Streptococcus; s__Streptococcus_salivarius;	2235	5622
k__Bacteria; p__Firmicutes; c__Clostridia; o__Lachnospirales; f__Lachnospiraceae; g__Lachnospiraceae_NK4A136_group; s__unclassified_Lachnospiraceae_NK4A136_group;	13	3883
k__Bacteria; p__Proteobacteria; c__Gammaproteobacteria; o__Enterobacterales; f__Pasteurellaceae; g__Haemophilus; s__Haemophilus_parainfluenzae;	20740	3762
k__Bacteria; p__Firmicutes; c__Negativicutes; o__Veillonellales_Selenomonadales; f__Veillonellaceae; g__Dialister; s__Dialister_invisus;	105	1625
k__Bacteria; p__Firmicutes; c__Negativicutes; o__Veillonellales_Selenomonadales; f__Veillonellaceae; g__Veillonella; s__Veillonella_dispar;	12162	1542
k__Bacteria; p__Proteobacteria; c__Gammaproteobacteria; o__Enterobacterales; f__Enterobacteriaceae; g__Klebsiella; s__Klebsiella_pneumoniae;	12	818
k__Bacteria; p__Firmicutes; c__Clostridia; o__Clostridia_UCG_014; f__unclassified_Clostridia_UCG_014; g__unclassified_Clostridia_UCG_014; s__unclassified_Clostridia_UCG_014;	148	734
k__Bacteria; p__Firmicutes; c__Bacilli; o__Lactobacillales; f__Streptococcaceae; g__Streptococcus; s__Streptococcus_parasanguinis;	747	692
k__Bacteria; p__Firmicutes; c__Clostridia; o__Lachnospirales; f__Lachnospiraceae; g__unclassified_Lachnospiraceae; s__unclassified_Lachnospiraceae;	24	365
k__Bacteria; p__Firmicutes; c__Negativicutes; o__Veillonellales_Selenomonadales; f__Veillonellaceae; g__Veillonella; s__Veillonella_parvula;	1650	254
k__Bacteria; p__Firmicutes; c__Bacilli; o__Lactobacillales; f__Carnobacteriaceae; g__Granulicatella; s__Granulicatella_adiacens;	5824	240
k__Bacteria; p__Patescibacteria; c__Saccharimonadia; o__Saccharimonadales; f__Saccharimonadaceae; g__TM7x; s__Candidatus_Nanosynbacter_lyticus;	1394	147
k__Bacteria; p__Firmicutes; c__Bacilli; o__Lactobacillales; f__Streptococcaceae; g__Streptococcus; s__Streptococcus_mitis;	148229	134
k__Bacteria; p__Actinobacteriota; c__Actinobacteria; o__Micrococcales; f__Micrococcaceae; g__Rothia;	1978	93

s__Rothia_dentocariosa;		
k__Bacteria; p__Firmicutes; c__Negativicutes; o__Veillonellales_Selenomonadales; f__Veillonellaceae; g__Veillonella; s__Veillonella_atypica;	153	77
k__Bacteria; p__Firmicutes; c__Bacilli; o__Erysipelotrichales; f__Erysipelotrichaceae; g__Solobacterium; s__Solobacterium_moorei;	309	76
k__Bacteria; p__Proteobacteria; c__Gammaproteobacteria; o__Burkholderiales; f__Burkholderiales_Incertae_Sedis; g__2013Ark19i; s__2013Ark19i_bacterium_2013Ark19i;	325	73
k__Bacteria; p__Firmicutes; c__Bacilli; o__Lactobacillales; f__Streptococcaceae; g__Streptococcus; s__Streptococcus_rubneri;	259	72
k__Bacteria; p__Firmicutes; c__Bacilli; o__Lactobacillales; f__Streptococcaceae; g__Streptococcus; s__Streptococcus_infantis;	980	60
k__Bacteria; p__Firmicutes; c__Bacilli; o__Lactobacillales; f__Streptococcaceae; g__Streptococcus; s__Pseudomonas_aeruginosa;	90	60
k__Bacteria; p__Firmicutes; c__Bacilli; o__Lactobacillales; f__Streptococcaceae; g__Streptococcus; s__Streptococcus_australis;	119	53
k__Bacteria; p__Firmicutes; c__Bacilli; o__Lactobacillales; f__Lactobacillaceae; g__Limosilactobacillus; s__Limosilactobacillus_fermentum;	14	43
k__Bacteria; p__Firmicutes; c__Bacilli; o__Lactobacillales; f__Streptococcaceae; g__Streptococcus; s__Streptococcus_oralis;	1956	41
k__Bacteria; p__Firmicutes; c__Bacilli; o__Lactobacillales; f__Streptococcaceae; g__Streptococcus; s__Streptococcus_sanguinis;	5869	38
k__Bacteria; p__Firmicutes; c__Bacilli; o__Lactobacillales; f__Aerococcaceae; g__Abiotrophia; s__Abiotrophia_defectiva;	2234	29
k__Bacteria; p__Proteobacteria; c__Gammaproteobacteria; o__Enterobacterales; f__Pasteurellaceae; g__Haemophilus; s__Haemophilus_sputorum;	446	25
k__Bacteria; p__Patescibacteria; c__Saccharimonadia; o__Saccharimonadales; f__uncultured_Candidatus_Saccharibacteria_bacterium; g__uncultured_Candidatus_Saccharibacteria_bacterium; s__uncultured_Candidatus_Saccharibacteria_bacterium;	1304	24
k__Bacteria; p__Patescibacteria; c__Saccharimonadia; o__Saccharimonadales; f__Saccharimonadaceae; g__TM7a; s__candidate_division_TM7_single_cell_isolate_TM7a;	296	22
k__Bacteria; p__Firmicutes; c__Bacilli; o__Staphylococcales; f__Gemellaceae; g__Gemella; s__Gemella_haemolysans;	27414	20

k__Bacteria; p__Patescibacteria; c__Saccharimonadia; o__Saccharimonadales; f__Saccharimonadaceae; g__uncultured_Candidatus_Saccharibacteria_bacterium; s__uncultured_Candidatus_Saccharibacteria_bacterium;	73	20
k__Bacteria; p__Firmicutes; c__Bacilli; o__Staphylococcales; f__Gemellaceae; g__Gemella; s__Gemella_sanguinis;	624	19
k__Bacteria; p__Firmicutes; c__Bacilli; o__Lactobacillales; f__Streptococcaceae; g__Streptococcus; s__Streptococcus_thermophilus;	161	18
k__Bacteria; p__Proteobacteria; c__Gammaproteobacteria; o__Enterobacterales; f__Pasteurellaceae; g__Aggregatibacter; s__Aggregatibacter_aphrophilus;	389	14
k__Bacteria; p__Firmicutes; c__Bacilli; o__Lactobacillales; f__Streptococcaceae; g__Streptococcus; s__Streptococcus_genomosp._C4;	2027	13
k__Bacteria; p__Firmicutes; c__Bacilli; o__Lactobacillales; f__Streptococcaceae; g__Streptococcus; s__Streptococcus_anginosus;	90	12
k__Bacteria; p__Proteobacteria; c__Gammaproteobacteria; o__Enterobacterales; f__Pasteurellaceae; g__Actinobacillus; s__Haemophilus_paraphrohaemolyticus_HK411;	694	10
k__Bacteria; p__Firmicutes; c__Bacilli; o__Lactobacillales; f__Streptococcaceae; g__Streptococcus; s__Streptococcus_intermedius;	24	10

Supplementary Table 6.Analysis of differences between gut and skin microbiomes.

taxonomy	Gut (abundances)	Skin (abundances)
k__Bacteria; p__Firmicutes; c__Bacilli; o__Staphylococcales; f__Staphylococcaceae; g__Staphylococcus; s__Staphylococcus_aureus;	19	27542
k__Bacteria; p__Firmicutes; c__Bacilli; o__Lactobacillales; f__Streptococcaceae; g__Streptococcus; s__Streptococcus_mitis;	134	19443
k__Bacteria; p__Firmicutes; c__Clostridia; o__Oscillospirales; f__Ruminococcaceae; g__Faecalibacterium; s__Faecalibacterium_prausnitzii;	28707	5836
k__Bacteria; p__Proteobacteria; c__Gammaproteobacteria; o__Burkholderiales; f__Burkholderiales_Incertae_Sedis; g__2013Ark19i; s__2013Ark19i_bacterium_2013Ark19i;	73	5338
k__Bacteria; p__Proteobacteria; c__Gammaproteobacteria; o__Enterobacterales; f__Pasteurellaceae; g__Haemophilus;	3762	3953

s__Haemophilus_parainfluenzae;		
k__Bacteria; p__Bacteroidota; c__Bacteroidia; o__Bacteroidales; f__Prevotellaceae; g__Prevotella_9; s__Prevotella_copri;	4836	3018
k__Bacteria; p__Proteobacteria; c__Gammaproteobacteria; o__Enterobacterales; f__Enterobacteriaceae; g__Escherichia_Shigella; s__Escherichia_coli;	23987	2944
k__Bacteria; p__Proteobacteria; c__Gammaproteobacteria; o__Enterobacterales; f__Enterobacteriaceae; g__Klebsiella; s__Klebsiella_pneumoniae;	818	2906
k__Bacteria; p__Firmicutes; c__Bacilli; o__Staphylococcales; f__Gemellaceae; g__Gemella; s__Gemella_haemolysans;	20	2133
k__Bacteria; p__Bacteroidota; c__Bacteroidia; o__Bacteroidales; f__Bacteroidaceae; g__Bacteroides; s__Bacteroides_fragilis;	14306	2090
k__Bacteria; p__Firmicutes; c__Bacilli; o__Lactobacillales; f__Streptococcaceae; g__Streptococcus; s__Streptococcus_salivarius;	5622	1666
k__Bacteria; p__Firmicutes; c__Bacilli; o__Lactobacillales; f__Streptococcaceae; g__Streptococcus; s__Streptococcus_sanguinis;	38	1645
k__Bacteria; p__Firmicutes; c__Bacilli; o__Lactobacillales; f__Carnobacteriaceae; g__Granulicatella; s__Granulicatella_adiacens;	240	1470
k__Bacteria; p__Actinobacteriota; c__Actinobacteria; o__Bifidobacteriales; f__Bifidobacteriaceae; g__Bifidobacterium; s__Bifidobacterium_pseudocatenulatum;	5820	1225
k__Bacteria; p__Firmicutes; c__Negativicutes; o__Acidaminococcales; f__Acidaminococcaceae; g__Phascolarctobacterium; s__Phascolarctobacterium_faecium;	1301	1132
k__Bacteria; p__Firmicutes; c__Bacilli; o__Lactobacillales; f__Enterococcaceae; g__Enterococcus; s__Enterococcus_faecium;	38	1128
k__Bacteria; p__Proteobacteria; c__Gammaproteobacteria; o__Pseudomonadales; f__Moraxellaceae; g__Acinetobacter; s__Acinetobacter_lwoffii;	3932	1114
k__Bacteria; p__Firmicutes; c__Negativicutes; o__Veillonellales_Selenomonadales; f__Veillonellaceae; g__Veillonella; s__Veillonella_dispar;	1542	1054
k__Bacteria; p__Firmicutes; c__Clostridia; o__Lachnospirales; f__Lachnospiraceae; g__Coprococcus; s__Coprococcus_eutactus;	263	1048
k__Bacteria; p__Verrucomicrobiota; c__Verrucomicrobiae; o__Verrucomicrobiales; f__Akkermansiaceae; g__Akkermansia; s__Akkermansia_muciniphila;	8810	1003
k__Bacteria; p__Firmicutes; c__Bacilli; o__Lactobacillales; f__Aerococcaceae; g__Abiotrophia; s__Abiotrophia_defectiva;	29	964
k__Bacteria; p__Firmicutes; c__Clostridia; o__Oscillospirales;	8377	958

f__Ruminococcaceae; g__Subdoligranulum; s__Faecalibacterium_prausnitzii;		
k__Bacteria; p__Firmicutes; c__Clostridia; o__Lachnospirales; f__Lachnospiraceae; g__Agathobacter; s__[Eubacterium]_rectale;	2223	939
k__Bacteria; p__Firmicutes; c__Clostridia; o__Oscillospirales; f__[Eubacterium]_coprostanoligenes_group; g__unclassified_[Eubacterium]_coprostanoligenes_group; s__unclassified_[Eubacterium]_coprostanoligenes_group;	2264	777
k__Bacteria; p__Bacteroidota; c__Bacteroidia; o__Bacteroidales; f__Bacteroidaceae; g__Bacteroides; s__Bacteroides_ovatus;	2872	751
k__Bacteria; p__Actinobacteriota; c__Actinobacteria; o__Micrococcales; f__Micrococcaceae; g__Rothia; s__Rothia_dentocariosa;	93	739
k__Bacteria; p__Proteobacteria; c__Gammaproteobacteria; o__Enterobacterales; f__Enterobacteriaceae; g__Enterobacter; s__Enterobacter_cloacae;	61	699
k__Bacteria; p__Bacteroidota; c__Bacteroidia; o__Bacteroidales; f__Bacteroidaceae; g__Bacteroides; s__Bacteroides_uniformis;	3784	674
k__Bacteria; p__Firmicutes; c__Bacilli; o__Erysipelotrichales; f__Erysipelatoclostridiaceae; g__Erysipelotrichaceae_UCG_003; s__Faecalibacillus_intestinalis;	1232	569
k__Bacteria; p__Firmicutes; c__Bacilli; o__Lactobacillales; f__Streptococcaceae; g__Streptococcus; s__Streptococcus_parasanguinis;	692	529
k__Bacteria; p__Firmicutes; c__Bacilli; o__Lactobacillales; f__Streptococcaceae; g__Streptococcus; s__Streptococcus_oralis;	41	516
k__Bacteria; p__Bacteroidota; c__Bacteroidia; o__Bacteroidales; f__Tannerellaceae; g__Parabacteroides; s__Parabacteroides_merdae;	373	485
k__Bacteria; p__Firmicutes; c__Clostridia; o__Oscillospirales; f__Ruminococcaceae; g__Faecalibacterium; s__Ruminococcaceae_bacterium;	579	464
k__Bacteria; p__Firmicutes; c__Clostridia; o__Lachnospirales; f__Lachnospiraceae; g__Lachnospiraceae_NK4A136_group; s__unclassified_Lachnospiraceae_NK4A136_group;	3883	452
k__Bacteria; p__Firmicutes; c__Negativicutes; o__Veillonellales_Selenomonadales; f__Veillonellaceae; g__Dialister; s__unclassified_Dialister;	448	448
k__Bacteria; p__Firmicutes; c__Clostridia; o__Lachnospirales; f__Lachnospiraceae; g__[Eubacterium]_hallii_group; s__[Eubacterium]_hallii;	453	436
k__Bacteria; p__Bacteroidota; c__Bacteroidia; o__Bacteroidales; f__Bacteroidaceae; g__Bacteroides; s__Bacteroides_vulgatus;	5361	417
k__Bacteria; p__Bacteroidota; c__Bacteroidia; o__Bacteroidales;	236	353

f__Prevotellaceae; g__Prevotella_9; s__Prevotellaceae_bacterium;		
k__Bacteria; p__Proteobacteria; c__Gammaproteobacteria; o__Enterobacterales; f__Enterobacteriaceae; g__Klebsiella; s__Klebsiella_oxytoca;	320	351
k__Bacteria; p__Firmicutes; c__Clostridia; o__Lachnospirales; f__Lachnospiraceae; g__Blautia; s__Blautia_wexlerae;	648	346
k__Bacteria; p__Bacteroidota; c__Bacteroidia; o__Bacteroidales; f__Rikenellaceae; g__Alistipes; s__Alistipes_finegoldii;	144	344
k__Bacteria; p__Firmicutes; c__Bacilli; o__Lactobacillales; f__Streptococcaceae; g__Streptococcus; s__Streptococcus_genomosp._C4;	13	343
k__Bacteria; p__Firmicutes; c__Negativicutes; o__Veillonellales_Selenomonadales; f__Selenomonadaceae; g__Megamonas; s__Megamonas_funiformis;	8082	334
k__Bacteria; p__Firmicutes; c__Bacilli; o__Lactobacillales; f__Streptococcaceae; g__Streptococcus; s__Streptococcus_infantis;	60	314
k__Bacteria; p__Firmicutes; c__Negativicutes; o__Veillonellales_Selenomonadales; f__Veillonellaceae; g__Veillonella; s__Veillonella_parvula;	254	301
k__Bacteria; p__Firmicutes; c__Clostridia; o__Oscillospirales; f__Ruminococcaceae; g__Faecalibacterium; s__Faecalibacterium_sp.;	1231	296
k__Bacteria; p__Firmicutes; c__Clostridia; o__Lachnospirales; f__Lachnospiraceae; g__[Ruminococcus]_torques_group; s__[Ruminococcus]_torques;	419	286
k__Bacteria; p__Bacteroidota; c__Bacteroidia; o__Bacteroidales; f__Bacteroidaceae; g__Bacteroides; s__Phocaeicola_vulgatus;	5230	284
k__Bacteria; p__Firmicutes; c__Clostridia; o__Lachnospirales; f__Lachnospiraceae; g__Fusicatenibacter; s__Fusicatenibacter_saccharivorans;	648	275
k__Bacteria; p__Firmicutes; c__Clostridia; o__Lachnospirales; f__Lachnospiraceae; g__Agathobacter; s__Eubacterium_ramulus;	33	257
k__Bacteria; p__Firmicutes; c__Clostridia; o__Peptostreptococcales_Tissierellales; f__Peptostreptococcaceae; g__Terrisporobacter; s__Terrisporobacter_glycolicus;	38	253
k__Bacteria; p__Proteobacteria; c__Gammaproteobacteria; o__Burkholderiales; f__Sutterellaceae; g__Parasutterella; s__Parasutterella_excrementihominis;	791	243
k__Bacteria; p__Firmicutes; c__Clostridia; o__Lachnospirales; f__Lachnospiraceae; g__Anaerostipes; s__Anaerostipes_hadrus;	1184	237
k__Bacteria; p__Proteobacteria; c__Gammaproteobacteria; o__Enterobacterales; f__Enterobacteriaceae; g__Enterobacter; s__Klebsiella_oxytoca;	126	234
k__Bacteria; p__Bacteroidota; c__Bacteroidia; o__Bacteroidales;	2598	233

f__Bacteroidaceae; g__Bacteroides; s__Phocaeicola_dorei;		
k__Bacteria; p__Firmicutes; c__Negativicutes; o__Veillonellales_Selenomonadales; f__Veillonellaceae; g__Dialister; s__Dialister_invisus;	1625	229
k__Bacteria; p__Firmicutes; c__Clostridia; o__Lachnospirales; f__Lachnospiraceae; g__Blautia; s__Ruminococcus_sp._Marseille_P328;	420	220
k__Bacteria; p__Firmicutes; c__Clostridia; o__Lachnospirales; f__Lachnospiraceae; g__Blautia; s__Blautia_obeum;	312	211
k__Bacteria; p__Patescibacteria; c__Saccharimonadia; o__Saccharimonadales; f__uncultured_Candidatus_Saccharibacteria_bacterium; g__uncultured_Candidatus_Saccharibacteria_bacterium; s__uncultured_Candidatus_Saccharibacteria_bacterium;	24	202
k__Bacteria; p__Firmicutes; c__Clostridia; o__Lachnospirales; f__Lachnospiraceae; g__unclassified_Lachnospiraceae; s__unclassified_Lachnospiraceae;	365	200
k__Bacteria; p__Actinobacteriota; c__Actinobacteria; o__Bifidobacteriales; f__Bifidobacteriaceae; g__Bifidobacterium; s__Bifidobacterium_longum;	246	197
k__Bacteria; p__Firmicutes; c__Clostridia; o__Oscillospirales; f__Ruminococcaceae; g__Ruminococcus; s__Ruminococcus_bicirculans;	148	195
k__Bacteria; p__Bacteroidota; c__Bacteroidia; o__Bacteroidales; f__Rikenellaceae; g__Alistipes; s__Alistipes_putredinis;	560	192
k__Bacteria; p__Bacteroidota; c__Bacteroidia; o__Bacteroidales; f__Bacteroidaceae; g__Bacteroides; s__Phocaeicola_plebeius;	1002	187
k__Bacteria; p__Firmicutes; c__Clostridia; o__Clostridia_UCG_014; f__uncultured_rumen_bacterium; g__uncultured_rumen_bacterium; s__uncultured_rumen_bacterium;	267	182
k__Bacteria; p__Firmicutes; c__Bacilli; o__Lactobacillales; f__Enterococcaceae; g__Enterococcus; s__Enterococcus_avium;	222	180
k__Bacteria; p__Firmicutes; c__Clostridia; o__Oscillospirales; f__Oscillospiraceae; g__UCG_005; s__unclassified_UCG_005;	95	175
k__Bacteria; p__Bacteroidota; c__Bacteroidia; o__Bacteroidales; f__Prevotellaceae; g__Prevotella_9; s__Trichuris_trichiura__human_whipworm;	3339	173
k__Bacteria; p__Proteobacteria; c__Gammaproteobacteria; o__Enterobacterales; f__Pasteurellaceae; g__Haemophilus; s__Haemophilus_sputorum;	25	170
k__Bacteria; p__Firmicutes; c__Clostridia; o__Clostridiales; f__Clostridiaceae; g__Clostridium_sensu_stricto_18; s__Clostridium_sporogenes;	933	155

k__Bacteria; p__Bacteroidota; c__Bacteroidia; o__Bacteroidales; f__Tannerellaceae; g__Parabacteroides; s__Parabacteroides_distasonis;	2282	144
k__Bacteria; p__Firmicutes; c__Clostridia; o__Oscillospirales; f__Ruminococcaceae; g__Ruminococcus; s__Ruminococcus_bromii;	1685	144
k__Bacteria; p__Bacteroidota; c__Bacteroidia; o__Bacteroidales; f__Bacteroidaceae; g__Bacteroides; s__Bacteroides_cellulosilyticus;	1248	143
k__Bacteria; p__Firmicutes; c__Clostridia; o__Oscillospirales; f__Ruminococcaceae; g__Subdoligranulum; s__Ruminococcaceae_bacterium;	489	143
k__Bacteria; p__Proteobacteria; c__Gammaproteobacteria; o__Enterobacterales; f__Pasteurellaceae; g__Actinobacillus; s__Haemophilus_paraphrohaemolyticus_HK411;	10	142
k__Bacteria; p__Firmicutes; c__Clostridia; o__Lachnospirales; f__Lachnospiraceae; g__Lachnoclostridium; s__unclassified_Lachnoclostridium;	1393	141
k__Bacteria; p__Firmicutes; c__Clostridia; o__Lachnospirales; f__Lachnospiraceae; g__CAG_56; s__unclassified_CAG_56;	143	133
k__Bacteria; p__Firmicutes; c__Clostridia; o__Lachnospirales; f__Lachnospiraceae; g__Dorea; s__Dorea_longicatena;	333	131
k__Bacteria; p__Firmicutes; c__Negativicutes; o__Veillonellales_Selenomonadales; f__Veillonellaceae; g__Dialister; s__Dialister_succinatiphilus;	154	127
k__Bacteria; p__Bacteroidota; c__Bacteroidia; o__Bacteroidales; f__Bacteroidaceae; g__Bacteroides; s__Bacteroides_stercoris;	266	124
k__Bacteria; p__Firmicutes; c__Bacilli; o__Staphylococcales; f__Gemellaceae; g__Gemella; s__Gemella_sanguinis;	19	124
k__Bacteria; p__Proteobacteria; c__Gammaproteobacteria; o__Enterobacterales; f__Enterobacteriaceae; g__Enterobacter; s__Enterobacter_bugandensis;	794	123
k__Bacteria; p__Proteobacteria; c__Gammaproteobacteria; o__Enterobacterales; f__Pasteurellaceae; g__Aggregatibacter; s__Aggregatibacter_aphrophilus;	14	122
k__Bacteria; p__Firmicutes; c__Bacilli; o__Lactobacillales; f__Streptococcaceae; g__Streptococcus; s__Streptococcus_thermophilus;	18	120
k__Bacteria; p__Firmicutes; c__Clostridia; o__Lachnospirales; f__Lachnospiraceae; g__Blautia; s__Blautia_massiliensis;	110	119
k__Bacteria; p__Firmicutes; c__Clostridia; o__Oscillospirales; f__Oscillospiraceae; g__NK4A214_group; s__unclassified_NK4A214_group;	45	118
k__Bacteria; p__Firmicutes; c__Bacilli; o__RF39;	610	116

f__uncultured_rumen_bacterium; g__uncultured_rumen_bacterium; s__uncultured_rumen_bacterium;		
k__Bacteria; p__Firmicutes; c__Clostridia; o__Lachnospirales; f__Lachnospiraceae; g__Dorea; s__Dorea_formicigenerans;	389	116
k__Bacteria; p__Firmicutes; c__Clostridia; o__Peptostreptococcales_Tissierellales; f__Peptostreptococcaceae; g__Romboutsia; s__Romboutsia_timonensis;	1143	112
k__Bacteria; p__Firmicutes; c__Clostridia; o__Clostridiales; f__Clostridiaceae; g__Clostridium_sensu_stricto_1; s__Clostridium_disporicum;	988	112
k__Bacteria; p__Firmicutes; c__Clostridia; o__Clostridia_UCG_014; f__unclassified_Clostridia_UCG_014; g__unclassified_Clostridia_UCG_014; s__unclassified_Clostridia_UCG_014;	734	100
k__Bacteria; p__Actinobacteriota; c__Actinobacteria; o__Bifidobacteriales; f__Bifidobacteriaceae; g__Bifidobacterium; s__Bifidobacterium_adolescentis;	234	100
k__Bacteria; p__Firmicutes; c__Clostridia; o__Oscillospirales; f__Ruminococcaceae; g__UBA1819; s__Ruthenibacterium_lactatiformans;	154	98
k__Bacteria; p__Firmicutes; c__Clostridia; o__Christensenellales; f__Christensenellaceae; g__Christensenellaceae_R_7_group; s__unclassified_Christensenellaceae_R_7_group;	1205	93
k__Bacteria; p__Firmicutes; c__Clostridia; o__Lachnospirales; f__Lachnospiraceae; g__Blautia; s__Lachnospiraceae_bacterium;	25	91
k__Bacteria; p__Firmicutes; c__Clostridia; o__Oscillospirales; f__Oscillospiraceae; g__UCG_002; s__unclassified_UCG_002;	123	88
k__Bacteria; p__Firmicutes; c__Clostridia; o__Peptostreptococcales_Tissierellales; f__Family_XI; g__Ezakiella; s__[Bacteroides]_coagulans;	21	88
k__Bacteria; p__Firmicutes; c__Clostridia; o__Monoglobales; f__Monoglobaceae; g__Monoglobus; s__uncultured_Clostridium_sp.;	965	77
k__Bacteria; p__Bacteroidota; c__Bacteroidia; o__Bacteroidales; f__Bacteroidaceae; g__Bacteroides; s__Bacteroides_caccae;	294	77
k__Bacteria; p__Firmicutes; c__Clostridia; o__Lachnospirales; f__Lachnospiraceae; g__[Ruminococcus]_torques_group; s__Ruminococcus_lactaris;	178	75
k__Bacteria; p__Fusobacteriota; c__Fusobacteriia; o__Fusobacteriales; f__Fusobacteriaceae; g__Cetobacterium; s__Cetobacterium_somerae;	3885	74
k__Bacteria; p__Firmicutes; c__Clostridia; o__Peptostreptococcales_Tissierellales; f__Peptostreptococcaceae;	147	74

g__Paraclostridium; s__Paraclostridium_bifermentans;		
k__Bacteria; p__Firmicutes; c__Clostridia; o__Oscillospirales; f__Oscillospiraceae; g__UCG_002; s__UCG_002_bacterium_P2C1;	61	74
k__Bacteria; p__Firmicutes; c__Clostridia; o__Lachnospirales; f__Lachnospiraceae; g__[Ruminococcus]_gnavus_group; s__[Ruminococcus]_gnavus;	1662	73
k__Bacteria; p__Firmicutes; c__Clostridia; o__Peptostreptococcales_Tissierellales; f__Peptostreptococcaceae; g__Intestinibacter; s__Intestinibacter_bartlettii;	259	73
k__Bacteria; p__Firmicutes; c__Clostridia; o__Peptostreptococcales_Tissierellales; f__Peptostreptococcaceae; g__Terrisporobacter; s__Terrisporobacter_mayombei;	40	72
k__Bacteria; p__Firmicutes; c__Clostridia; o__Oscillospirales; f__Ruminococcaceae; g__unclassified_Ruminococcaceae; s__unclassified_Ruminococcaceae;	106	71
k__Bacteria; p__Firmicutes; c__Bacilli; o__Lactobacillales; f__Streptococcaceae; g__Streptococcus; s__Streptococcus_australis;	53	70
k__Bacteria; p__Firmicutes; c__Clostridia; o__Oscillospirales; f__Ruminococcaceae; g__[Eubacterium]_siraeum_group; s__[Eubacterium]_siraeum;	1219	69
k__Bacteria; p__Firmicutes; c__Clostridia; o__Lachnospirales; f__Lachnospiraceae; g__Sellimonas; s__Sellimonas_intestinalis;	66	69
k__Bacteria; p__Firmicutes; c__Clostridia; o__Lachnospirales; f__Lachnospiraceae; g__Coprococcus; s__Coprococcus_comes;	325	68
k__Bacteria; p__Bacteroidota; c__Bacteroidia; o__Bacteroidales; f__Bacteroidaceae; g__Bacteroides; s__Bacteroides_thetaiotaomicron;	1257	67
k__Bacteria; p__Firmicutes; c__Clostridia; o__Peptostreptococcales_Tissierellales; f__Anaerovoracaceae; g__Family_XIII_AD3011_group; s__unclassified_Family_XIII_AD3011_group;	79	67
k__Bacteria; p__Firmicutes; c__Clostridia; o__Lachnospirales; f__Lachnospiraceae; g__Blautia; s__Blautia_luti;	51	67
k__Bacteria; p__Firmicutes; c__Clostridia; o__Lachnospirales; f__Lachnospiraceae; g__Roseburia; s__Roseburia_faecis;	492	66
k__Bacteria; p__Firmicutes; c__Clostridia; o__Oscillospirales; f__Ruminococcaceae; g__CAG_352; s__Anaeromassilibacillus_sp._Marseille_P3876;	830	65
k__Bacteria; p__Firmicutes; c__Clostridia; o__Clostridiales; f__Clostridiaceae; g__Clostridium_sensu_stricto_18; s__Clostridium_botulinum;	801	60
k__Bacteria; p__Firmicutes; c__Clostridia; o__Oscillospirales;	332	60

f__Butyricicoccaceae; g__Butyricicoccus; s__Agathobaculum_butyriciproducens;		
k__Bacteria; p__Firmicutes; c__Clostridia; o__Lachnospirales; f__Lachnospiraceae; g__[Ruminococcus]_gauvreauii_group; s__Lachnospiraceae_bacterium;	35	60
k__Bacteria; p__Firmicutes; c__Bacilli; o__Erysipelotrichales; f__Erysipelotrichaceae; g__Holdemanella; s__Holdemanella_biformis;	387	59
k__Bacteria; p__Firmicutes; c__Clostridia; o__Oscillospirales; f__Ruminococcaceae; g__Faecalibacterium; s__unclassified_Faecalibacterium;	112	58
k__Bacteria; p__Bacteroidota; c__Bacteroidia; o__Bacteroidales; f__Bacteroidaceae; g__Bacteroides; s__unclassified_Bacteroides;	495	50
k__Bacteria; p__Firmicutes; c__Clostridia; o__Clostridiales; f__Clostridiaceae; g__Clostridium_sensu_stricto_1; s__Clostridium_perfringens;	44	50
k__Bacteria; p__Firmicutes; c__Bacilli; o__Erysipelotrichales; f__Erysipelotrichaceae; g__[Clostridium]_innocuum_group; s__[Clostridium]_innocuum;	118	49
k__Bacteria; p__Firmicutes; c__Bacilli; o__Lactobacillales; f__Streptococcaceae; g__Streptococcus; s__Pseudomonas_aeruginosa;	60	49
k__Bacteria; p__Desulfobacterota; c__Desulfovibrionia; o__Desulfovibrionales; f__Desulfovibrionaceae; g__Bilophila; s__Bilophila_wadsworthia;	44	49
k__Bacteria; p__Firmicutes; c__Clostridia; o__Oscillospirales; f__Oscillospiraceae; g__unclassified_Oscillospiraceae; s__unclassified_Oscillospiraceae;	130	48
k__Bacteria; p__Firmicutes; c__Bacilli; o__Erysipelotrichales; f__Erysipelotrichaceae; g__Solobacterium; s__Solobacterium_moorei;	76	48
k__Bacteria; p__Firmicutes; c__Clostridia; o__Lachnospirales; f__Lachnospiraceae; g__Blautia; s__Blautia_sp._Marseille_P3087;	13	47
k__Bacteria; p__Firmicutes; c__Bacilli; o__Lactobacillales; f__Streptococcaceae; g__Streptococcus; s__Streptococcus_anginosus;	12	47
k__Bacteria; p__Firmicutes; c__Clostridia; o__Lachnospirales; f__Lachnospiraceae; g__Lachnospiraceae_NK4A136_group; s__Lachnospiraceae_bacterium_GAM79;	141	46
k__Bacteria; p__Firmicutes; c__Bacilli; o__Erysipelotrichales; f__Erysipelatoclostridiaceae; g__Erysipelatoclostridium; s__Erysipelatoclostridium_ramosum;	876	44
k__Bacteria; p__Firmicutes; c__Clostridia; o__Lachnospirales; f__Lachnospiraceae; g__Lachnoclostridium;	13	44

s__Lachnospiraceae_bacterium;		
k__Bacteria; p__Bacteroidota; c__Bacteroidia; o__Bacteroidales; f__Rikenellaceae; g__Alistipes; s__Alistipes_shahii;	79	42
k__Bacteria; p__Firmicutes; c__Clostridia; o__Lachnospirales; f__Lachnospiraceae; g__[Eubacterium]_ruminantium_group; s__unclassified_[Eubacterium]_ruminantium_group;	40	39
k__Bacteria; p__Firmicutes; c__Clostridia; o__Lachnospirales; f__Lachnospiraceae; g__Coprococcus; s__Lachnospiraceae_bacterium;	19	39
k__Bacteria; p__Firmicutes; c__Clostridia; o__Lachnospirales; f__Lachnospiraceae; g__Lachnoclostridium; s__[Clostridium]_symbiosum;	10	39
k__Bacteria; p__Firmicutes; c__Clostridia; o__Eubacteriales; f__Eubacteriaceae; g__Eubacterium; s__Eubacterium_limosum;	20	38
k__Bacteria; p__Bacteroidota; c__Bacteroidia; o__Bacteroidales; f__Bacteroidaceae; g__Bacteroides; s__Phocaeicola_coprocola;	784	36
k__Bacteria; p__Firmicutes; c__Clostridia; o__Lachnospirales; f__Lachnospiraceae; g__Hungatella; s__Hungatella_hathewayi;	61	33
k__Bacteria; p__Actinobacteriota; c__Actinobacteria; o__Bifidobacteriales; f__Bifidobacteriaceae; g__Bifidobacterium; s__Bifidobacterium_breve;	47	33
k__Bacteria; p__Firmicutes; c__Bacilli; o__Lactobacillales; f__Streptococcaceae; g__Streptococcus; s__Streptococcus_rubneri;	72	32
k__Bacteria; p__Firmicutes; c__Clostridia; o__Oscillospirales; f__Oscillospiraceae; g__Colidextribacter; s__Clostridiales_bacterium_CCNA10;	10	32
k__Bacteria; p__Firmicutes; c__Clostridia; o__Lachnospirales; f__Lachnospiraceae; g__[Eubacterium]_eligens_group; s__[Eubacterium]_eligens;	1142	31
k__Bacteria; p__Firmicutes; c__Clostridia; o__Lachnospirales; f__Lachnospiraceae; g__[Eubacterium]_ventriosum_group; s__uncultured_Lachnospiraceae_bacterium;	111	31
k__Bacteria; p__Bacteroidota; c__Bacteroidia; o__Bacteroidales; f__Bacteroidaceae; g__Bacteroides; s__Bacteroides_nordii;	91	31
k__Bacteria; p__Proteobacteria; c__Gammaproteobacteria; o__Enterobacterales; f__Morganellaceae; g__Morganella; s__Morganella_morganii;	16	31
k__Bacteria; p__Firmicutes; c__Clostridia; o__Lachnospirales; f__Lachnospiraceae; g__Lachnoclostridium; s__Clostridium_fessum;	898	30
k__Bacteria; p__Proteobacteria; c__Gammaproteobacteria; o__Enterobacterales; f__Enterobacteriaceae; g__Citrobacter; s__Citrobacter_freundii;	771	30
k__Bacteria; p__Firmicutes; c__Negativicutes;	77	30

o__Veillonellales_Selenomonadales; f__Veillonellaceae; g__Veillonella; s__Veillonella_atypica;		
k__Bacteria; p__Firmicutes; c__Clostridia; o__Oscillospirales; f__Oscillospiraceae; g__Oscillibacter; s__Oscilibacter_massiliensis;	79	29
k__Bacteria; p__Firmicutes; c__Clostridia; o__Oscillospirales; f__Oscillospiraceae; g__UCG_003; s__Marseillibacter_massiliensis;	77	29
k__Bacteria; p__Firmicutes; c__Clostridia; o__Oscillospirales; f__Ruminococcaceae; g__Ruminococcus; s__Ruminococcus_callidus;	109	27
k__Bacteria; p__Firmicutes; c__Clostridia; o__Lachnospirales; f__Lachnospiraceae; g__Lachnospiraceae_FCS020_group; s__Lachnospiraceae_bacterium;	13	27
k__Bacteria; p__Firmicutes; c__Bacilli; o__Lactobacillales; f__Streptococcaceae; g__Streptococcus; s__Streptococcus_intermedius;	10	27
k__Bacteria; p__Firmicutes; c__Negativicutes; o__Veillonellales_Selenomonadales; f__Veillonellaceae; g__Megasphaera; s__unclassified_Megasphaera;	590	25
k__Bacteria; p__Firmicutes; c__Clostridia; o__Lachnospirales; f__Lachnospiraceae; g__Roseburia; s__Roseburia_inulinivorans;	339	25
k__Bacteria; p__Firmicutes; c__Clostridia; o__Oscillospirales; f__Ruminococcaceae; g__Ruminococcus; s__Ruminococcus_sp.;	322	24
k__Bacteria; p__Patescibacteria; c__Saccharimonadia; o__Saccharimonadales; f__Saccharimonadaceae; g__TM7a; s__candidate_division_TM7_single_cell_isolate_TM7a;	22	23
k__Bacteria; p__Firmicutes; c__Clostridia; o__Lachnospirales; f__Lachnospiraceae; g__Roseburia; s__Roseburia_hominis;	171	22
k__Bacteria; p__Bacteroidota; c__Bacteroidia; o__Bacteroidales; f__Rikenellaceae; g__Alistipes; s__Alistipes_indistinctus;	50	22
k__Bacteria; p__Firmicutes; c__Clostridia; o__Oscillospirales; f__Ruminococcaceae; g__Incertae_Sedis; s__[Clostridium]_leptum;	30	22
k__Bacteria; p__Patescibacteria; c__Saccharimonadia; o__Saccharimonadales; f__Saccharimonadaceae; g__TM7x; s__Candidatus_Nanosynbacter_lyticus;	147	21
k__Bacteria; p__Firmicutes; c__Clostridia; o__Lachnospirales; f__Lachnospiraceae; g__Fusicatenibacter; s__Lachnospiraceae_bacterium;	97	21
k__Bacteria; p__Firmicutes; c__Clostridia; o__Lachnospirales; f__Lachnospiraceae; g__Lachnospiraceae_ND3007_group; s__Roseburia_sp._1120;	41	21
k__Bacteria; p__Firmicutes; c__Clostridia; o__Lachnospirales;	40	21

f__Lachnospiraceae; g__[Ruminococcus]_torques_group; s__Mediterraneibacter_faecis;		
k__Bacteria; p__Firmicutes; c__Clostridia; o__Oscillospirales; f__Oscillospiraceae; g__Flavonifractor; s__Flavonifractor_plautii;	73	20
k__Bacteria; p__Firmicutes; c__Clostridia; o__Lachnospirales; f__Lachnospiraceae; g__Lachnospira; s__Lactobacillus_rogosae;	314	19
k__Bacteria; p__Firmicutes; c__Clostridia; o__Lachnospirales; f__Lachnospiraceae; g__Eisenbergiella; s__Eisenbergiella_tayi;	143	19
k__Bacteria; p__Firmicutes; c__Bacilli; o__Lactobacillales; f__Lactobacillaceae; g__Limosilactobacillus; s__Limosilactobacillus_fermentum;	43	19
k__Bacteria; p__Firmicutes; c__Clostridia; o__Lachnospirales; f__Lachnospiraceae; g__Lachnospiraceae_UCG_004; s__[Eubacterium]_hallii;	38	17
k__Bacteria; p__Bacteroidota; c__Bacteroidia; o__Bacteroidales; f__Barnesiellaceae; g__Barnesiella; s__Barnesiella_intestinihominis;	29	17
k__Bacteria; p__Firmicutes; c__Clostridia; o__Lachnospirales; f__Lachnospiraceae; g__Lachnoclostridium; s__Lachnoclostridium_sp._mt68;	23	17
k__Bacteria; p__Firmicutes; c__Clostridia; o__Oscillospirales; f__Ruminococcaceae; g__Subdoligranulum; s__unclassified_Subdoligranulum;	28	16
k__Bacteria; p__Proteobacteria; c__Gammaproteobacteria; o__Enterobacterales; f__Enterobacteriaceae; g__Enterobacter; s__Enterobacter_cancerogenus;	15	16
k__Bacteria; p__Firmicutes; c__Clostridia; o__Clostridiales; f__Clostridiaceae; g__Clostridium_sensu_stricto_1; s__unclassified_Clostridium_sensu_stricto_1;	13	16
k__Bacteria; p__Firmicutes; c__Clostridia; o__Lachnospirales; f__Lachnospiraceae; g__Roseburia; s__Roseburia_intestinalis;	328	15
k__Bacteria; p__Firmicutes; c__Clostridia; o__Lachnospirales; f__Lachnospiraceae; g__[Eubacterium]_ventriosum_group; s__unclassified_[Eubacterium]_ventriosum_group;	13	15
k__Bacteria; p__Firmicutes; c__Clostridia; o__Christensenellales; f__Christensenellaceae; g__Christensenellaceae_R_7_group; s__Clostridiales_bacterium_Marseille_P2846;	153	14
k__Bacteria; p__Bacteroidota; c__Bacteroidia; o__Bacteroidales; f__Prevotellaceae; g__Paraprevotella; s__Paraprevotella_clara;	85	14
k__Bacteria; p__Firmicutes; c__Bacilli; o__RF39; f__uncultured_Firmicutes_bacterium; g__uncultured_Firmicutes_bacterium; s__uncultured_Firmicutes_bacterium;	37	14
k__Bacteria; p__Patescibacteria; c__Saccharimonadia;	20	12

o__Saccharimonadales; f__Saccharimonadaceae; g__uncultured_Candidatus_Saccharibacteria_bacterium; s__uncultured_Candidatus_Saccharibacteria_bacterium;		
k__Bacteria; p__Firmicutes; c__Clostridia; o__Oscillospirales; f__Ruminococcaceae; g__Incertae_Sedis; s__Clostridiaceae_bacterium_DJF_LS40;	18	12
k__Bacteria; p__Firmicutes; c__Negativicutes; o__Veillonellales_Selenomonadales; f__Veillonellaceae; g__Dialister; s__Dialister_massiliensis;	1116	11
k__Bacteria; p__Firmicutes; c__Clostridia; o__Oscillospirales; f__Ruminococcaceae; g__Incertae_Sedis; s__unclassified_Incertae_Sedis;	134	10
k__Bacteria; p__Bacteroidota; c__Bacteroidia; o__Bacteroidales; f__Marinifilaceae; g__Odoribacter; s__Odoribacter_splanchnicus;	110	10
k__Bacteria; p__Firmicutes; c__Bacilli; o__Erysipelotrichales; f__Erysipelotrichaceae; g__Holdemania; s__Holdemania_filiformis;	98	10
k__Bacteria; p__Firmicutes; c__Clostridia; o__Peptostreptococcales_Tissierellales; f__Peptostreptococcaceae; g__Romboutsia; s__Romboutsia_lituseburensis;	12	10
