# Supplementary material for: Changes in oral, skin, and gut microbiota in children with atopic dermatitis: a case-control study
Source: Front Microbiol. 2024 Aug 15;15:1442126. doi: 10.3389/fmicb.2024.1442126 (PMC11358084; doi:10.3389/fmicb.2024.1442126)
Supplement: Supplementary file 5 [file Data_Sheet_5.PDF]

Supplementary Table 7.Correlation Analysis of Shared Microbiota, Differential Metabolites, and Metagenomic Data in Children with AD.

Microbes		Metabolites	Metagenes	KO function	Relation
	oral	Cyclic Urea			
Escherichia_coli	skin	6-Keto-prostaglandin E1	K03404	magnesium chelatase subunit D	positively
	gut	Nomifensine			
			K00313	electron transfer flavoprotein-quinone oxidoreductase	
			K00366	ferredoxin-nitrite reductase	
			K03404	magnesium chelatase subunit D	
	oral	L-alpha-Amino-1 H-pyrrole-1-hexanoic acid	K05606	methylmalonyl-CoA/ethylmalonyl-CoA epimerase	positively
			K16149	1,4-alpha-glucan branching enzyme	
			K19405	protein arginine kinase	
			K06409	stage V sporulation protein B	
			K00313	electron transfer flavoprotein-quinone oxidoreductase	
Faecalibacterium_prausnitzii			K00366	ferredoxin-nitrite reductase	
			K01751	diaminopropionate ammonia-lyase	
			K01848	methylmalonyl-CoA mutase, N-terminal domain	
			K01849	methylmalonyl-CoA mutase, C-terminal domain	
	skin	L-alpha-Amino-1 H-pyrrole-1-hexanoic acid	K03404	magnesium chelatase subunit D	positively
			K05606	methylmalonyl-CoA/ethylmalonyl-CoA epimerase	
			K16149	1,4-alpha-glucan branching enzyme	
			K19405	protein arginine kinase	
			K06409	stage V sporulation protein B	

			K07011	uncharacterized protein	
			K09477	citrate:succinate antiporter	
			K11102	proton glutamate symport protein	
			K00366	ferredoxin-nitrite reductase	
			K01751	diaminopropionate ammonia-lyase	
			K01848	methylmalonyl-CoA mutase, N-terminal domain	
			K01849	methylmalonyl-CoA mutase, C-terminal domain	
			K03404	magnesium chelatase subunit D	
	gut	2alpha-(3-Hydroxypropyl)-1 alpha	K05606	methylmalonyl-CoA/ethylmalonyl-CoA epimerase	negatively
			K07141	molybdenum cofactor cytidylyltransferase	
			K09477	citrate:succinate antiporter	
			K11102	proton glutamate symport protein	
			K16149	1,4-alpha-glucan branching enzyme	
			K19405	protein arginine kinase	
	oral	Dodecanedioic acid<br>Undecanedioic acid			
Gemella_haemolysans	skin	6-Keto-prostaglandin E1<br>beta-Bixin, Lyciumoside<br>Naematolone	K03404	magnesium chelatase subunit D	negatively
	gut	cis-Linoleic acid<br>Nomifensine			
			K00261	glutamate dehydrogenase (NAD(P)+)	
			K00284	glutamate synthase (ferredoxin)	
			K00313	electron transfer flavoprotein-quinone oxidoreductase	
Neisseria_mucosa	oral	Tris (2-ethylhexyl) phosphate			negatively
			K00366	ferredoxin-nitrite reductase	
			K00574	cyclopropane-fatty-acyl-phospholipid synthase	
			K00772	5'-methylthioadenosine phosphorylase	

		K01751	diaminopropionate ammonia-lyase
		K01848	methylmalonyl-CoA mutase, N-terminal domain
		K01849	methylmalonyl-CoA mutase, C-terminal domain
		K03404	magnesium chelatase subunit D
		K05606	methylmalonyl-CoA/ethylmalonyl-CoA epimerase
		K06409	stage V sporulation protein B
		K07005	uncharacterized protein
		K07402	xanthine dehydrogenase accessory factor
		K07101	uncharacterized protein
		K07141	molybdenum cofactor cytidylyltransferase
		K08325	NADP-dependent alcohol dehydrogenase
		K09477	citrate:succinate antiporter
		K09729	uncharacterized protein
		K08989	putative membrane protein
		K09775	uncharacterized protein
		K09807	uncharacterized protein
		K11102	proton glutamate symport protein
		K11709	manganese/zinc/iron transport system permease protein
		K15633	2,3-bisphosphoglycerate-independent phosphoglycerate mutase
		K16149	1,4-alpha-glucan branching enzyme
		K18333	L-fucose dehydrogenase
		K19221	cob(I)alamin adenosyltransferas
		K19405	protein arginine kinase
skin	22alpha-Hydroxy-campester	K00966	mannose-1-phosphate guanylyltransferase

		ol beta-Bixin, Lyciumoside	K03404	magnesium chelatase subunit D	
		PG(i-12:0/6 keto-PGF1alpha)	K09729	uncharacterized protein	
		Naematolone	K11709	protection of telomeres protein 1	
			K00313	electron transfer flavoprotein-quinone oxidoreductase	
			K00366	ferredoxin-nitrite reductase	
			K01751	diaminopropionate ammonia-lyase	
			K01848	methylmalonyl-CoA mutase, N-terminal domain	
			K01849	methylmalonyl-CoA mutase, C-terminal domain	
			K03404	magnesium chelatase subunit D	
			K05606	methylmalonyl-CoA/ethylmalonyl-CoA epimerase	
			K06409	stage V sporulation protein B	
		2alpha-(3-Hydroxypropyl)-1alpha	K07141	molybdenum cofactor cytidylyltransferase	
	gut	25-dihydroxy-19-norvitamin D3	K09477	citrate:succinate antiporter	positively
		Androsterone	K11102	proton glutamate symport protein	
			K16149	1,4-alpha-glucan branching enzyme	
			K19405	protein arginine kinase	
			K00266	glutamate synthase (NADPH/NADH) small chain	
			K00574	cyclopropane-fatty-acyl-phospholipid synthase	
			K00772	5'-methylthioadenosine phosphorylase	
			K07266	capsular polysaccharide export protein	
			K07588	LAO/AO transport system kinase	
			K08325	NADP-dependent alcohol dehydrogenase	
		cis-Linoleic acid	K03404	magnesium chelatase subunit D	negatively
Staphylococcus_aureus	oral	(16Z) -14-Hydroxydocos-16-enoylc	K00966	mannose-1-phosphate guanylyltransferase	negatively

		arnitine<br>3-Hydroxydodeca-6,9-dienoylcarnitine<br>Butyric acid, Cyclic Urea, Dodecanedioic acid<br>L-alpha-Amino-1H-pyrrole-1-hexanoic acid<br>Undecanedioic acid	K03404	magnesium chelatase subunit D	
	skin	(x)-2-Heptanol glucoside<br>17-hydroxylinolenic acid<br>alpha-Tocopherol<br>L-alpha-Amino-1H-pyrrole-1-hexanoic acid<br>PG(i-12:0/6 keto-PGF1alpha)	K03404	magnesium chelatase subunit D	
	gut	2alpha-(3-Hydroxypropyl)-1alpha 25-dihydroxy-19-norvitamin D3	K00313	electron transfer flavoprotein-quinone oxidoreductase	
			K01848	methylmalonyl-CoA mutase, N-terminal domain	
			K01849	methylmalonyl-CoA mutase, C-terminal domain	
			K03404	magnesium chelatase subunit D	
			K05606	methylmalonyl-CoA/ethylmalonyl-CoA epimerase	
			K06409	stage V sporulation protein B	
			K07588	LAO/AO transport system kinase	
			K09477	citrate:succinate antiporter	
			K11102	proton glutamate symport protein	
			K16149	1,4-alpha-glucan branching enzyme	
			K19405	protein arginine kinase	
Streptococcus_mitis	oral	Butyric acid, Dodecanedioic acid<br>Tris (2-ethylhexyl) phosphate<br>Undecanedioic acid	K03404	magnesium chelatase subunit D	negatively
	skin	22alpha-Hydroxy-campesterol<br>6-Keto-prostaglandin E1<br>beta-Bixin<br>Lyciumoside	K03404	magnesium chelatase subunit D	

		Naematolone<br>PG(i-12:0/6<br>keto-PGF1alpha)			
gut		cis-Linoleic acid<br>2alpha-(3-Hydroxypropyl)-1<br>alpha<br>25-dihydroxy-19-norvitamin<br>D3	K00313	electron transfer flavoprotein-quinone oxidoreductase	positively
			K03404	magnesium chelatase subunit D	
			K19405	protein arginine kinase	
			K16149	1,4-alpha-glucan branching enzyme	
			K07141	molybdenum cofactor cytidylyltransferase	
			K06409	stage V sporulation protein B	
			K11102	proton glutamate symport protein	
			K09477	citrate:succinate antiporter	
			K07588	LAO/AO transport system kinase	
			K05606	methylmalonyl-CoA/ethylmalonyl-CoA epimerase	
			K01848	methylmalonyl-CoA mutase, N-terminal domain	
			K01849	methylmalonyl-CoA mutase, C-terminal domain	
			K01751	diaminopropionate ammonia-lyase	
			K00366	ferredoxin-nitrite reductase	

Supplementary Table 8.Correlation analysis between differential strains, metabolites and functional genes.

Position	Microbes	Metabolites	Metagenes	KO function	Relation
	Cronobacter sakazakii	alpha-Tocopherol	K02529	LacI family transcriptional regulator, galactose operon repressor	negatively
skin	Human endogenous retrovirus W	2-Chlorohexadecanal<br>MG(0:0/20:5(5Z,8Z,11Z,14Z,17Z)/0:0)<br>Tetrofosmin	K00656	formate C-acetyltransferase	positively
			K01186	sialidase-1	
			K01390	IgA-specific metalloendopeptidase	
			K02030	polar amino acid transport system substrate-binding protein	
			K03111	single-strand DNA-binding protein	
			K03555	DNA mismatch repair protein MutS	

		K03696	ATP-dependent Clp protease ATP-binding subunit ClpC	
		K06148	ATP-binding cassette, subfamily C, bacterial	
		K01637	isocitrate lyase	
		K07052	CAAX protease family protein	
Mycobacterium tuberculosis	2-Chlorohexadecanal<br>MG(0:0/20:5(5Z,8Z,11Z,14Z,17Z)/0:0)	K01637	isocitrate lyase	positively
Streptococcus infantis	2-Chlorohexadecanal<br>MG(0:0/20:5(5Z,8Z,11Z,14Z,17Z)/0:0)<br>Tetrofosmin	K00656	formate C-acetyltransferase	negatively
		K01186	sialidase-1	
		K01390	IgA-specific metalloendopeptidase	
		K02030	polar amino acid transport system substrate-binding protein	
		K03111	single-strand DNA-binding protein	
		K03555	DNA mismatch repair protein MutS	
Streptococcus mitis	alpha-Tocopherol	K01186	sialidase-1	positively
		K03111	single-strand DNA-binding protein	
		K02529	LacI family transcriptional regulator, galactose operon repressor	
		K06180	23S rRNA pseudouridine1911/1915/1917 synthase	
		K07052	CAAX protease family protein	
Streptococcus oralis	2-Chlorohexadecanal<br>MG(0:0/20:5(5Z,8Z,11Z,14Z,17Z)/0:0)<br>Tetrofosmin	K00656	formate C-acetyltransferase	negatively
		K01186	sialidase-1	
		K02030	polar amino acid transport system substrate-binding protein	
		K07052	CAAX protease family protein	
		K06148	ATP-binding cassette, subfamily C, bacterial	
		K03763	DNA polymerase III subunit alpha, Gram-positive type	
		K03696	ATP-dependent Clp protease ATP-binding subunit ClpC	
		K03555	DNA mismatch repair protein MutS	
Streptococcus parasanguinis	alpha-Tocopherol	K03111	single-strand DNA-binding protein	positively
		K02529	LacI family transcriptional regulator, galactose operon repressor	
		K02029	polar amino acid transport system permease protein	
Streptococcus	alpha-Tocopherol	K06180	23S rRNA	positively

	pneumoniae			pseudouridine1911/1915/1917 synthase	
			K03111	single-strand DNA-binding protein	
			K02529	LacI family transcriptional regulator, galactose operon repressor	
			K01186	sialidase-1	
	Streptococcus pseudopneumoniae	alpha-Tocopherol	K03111	single-strand DNA-binding protein	positively
			K02529	LacI family transcriptional regulator, galactose operon repressor	
			K07052	CAAX protease family protein	
	Streptococcus sp.	alpha-Tocopherol	K02529	LacI family transcriptional regulator, galactose operon repressor	positively
			K06180	23S rRNA pseudouridine1911/1915/1917 synthase	
	Streptococcus sp. C300	alpha-Tocopherol	K02529	LacI family transcriptional regulator, galactose operon repressor	positively
	Streptococcus sp. CCuG 49591	2-Chlorohexadecanal	K00656	formate C-acetyltransferase	negatively
		MG(0:0/20:5(5Z,8Z,11Z,14Z,17Z)/0:0)	K01186	sialidase-1	
		Tetrofosmin	K01637	isocitrate lyase	
			K02030	polar amino acid transport system substrate-binding protein	
	Streptococcus_sp. DORA_10	alpha-Tocopherol	K02529	LacI family transcriptional regulator, galactose operon repressor	positively
			K06180	23S rRNA pseudouridine1911/1915/1917 synthase	
	Streptococcus sp. OH4692 COT 348	alpha-Tocopherol	K02029	polar amino acid transport system permease protein	positively
			K03111	single-strand DNA-binding protein	
			K02529	LacI family transcriptional regulator, galactose operon repressor	
			K06180	23S rRNA pseudouridine1911/1915/1917 synthase	
			K07052	CAAX protease family protein	
	Streptococcus sp. oral taxon 058	alpha-Tocopherol	K02529	LacI family transcriptional regulator, galactose operon repressor	positively
	taxon 058Streptococcus	alpha-Tocopherol	K02529	LacI family transcriptional regulator, galactose operon repressor	positively
oral	Fusobacterium_sp._oral_taxorn 370	Octadecanamide	K03110	fused signal recognition particle receptor	positively
			K12267	peptide methionine sulfoxide reductase msrA/msrB	
	Hyphomicrobiales_bacterium	2-Chlorohexadecanal	K00527	ribonucleoside-triphosphate reductase (thioredoxin)	positively

	Octadecanamide	K03110	fused signal recognition particle receptor	
		K12267	peptide methionine sulfoxide reductase msrA/msrB	
Kytococcus sedentarius	Octadecanamide	K03110	fused signal recognition particle receptor	positively
		K16787	energy-coupling factor transport system ATP-binding protein	
		K15738	ABC transport system ATP-binding/permease protein	
		K00527	ribonucleoside-triphosphate reductase (thioredoxin)	
Lachnoanaerobaculum umeaense	2-Chlorohexadecanal Octadecanamide	K03110	fused signal recognition particle receptor	positively
		K12267	peptide methionine sulfoxide reductase msrA/msrB	
		K16787	energy-coupling factor transport system ATP-binding protein	
		K16798	zinc finger protein GLI2	
		K15580	oligopeptide transport system substrate-binding protein	
		K12574	ribonuclease J	
		K11754	dihydrofolate synthase / folylpolyglutamate synthase	
		K15738	ABC transport system ATP-binding/permease protein	
		K16785	energy-coupling factor transport system permease protein	
		K16786	energy-coupling factor transport system ATP-binding protein	
	alpha-Tocopherol	K03110	fused signal recognition particle receptor	negatively
		K03555	DNA mismatch repair protein MutS	
		K05846	osmoprotectant transport system permease protein	
		K12267	peptide methionine sulfoxide reductase msrA/msrB	
		K15580	oligopeptide transport system substrate-binding protein	
		K16785	energy-coupling factor transport system permease protein	
Ligilactobacillus_murinus	2-Chlorohexadecanal Octadecanamide	K03110	fused signal recognition particle receptor	positively
		K12267	peptide methionine sulfoxide reductase msrA/msrB	
		K16785	energy-coupling factor transport system permease protein	
		K16787	energy-coupling factor transport system ATP-binding protein	

	Neisseria_sp. HMSC068C12	2-Chlorohexadecanal Octadecanamide Heptaethylene glycol monododecyl eteret Tetracosatetraenoyl carnitine	K03110	fused signal recognition particle receptor	positively
			K15580	oligopeptide transport system substrate-binding protein	
			K12267	peptide methionine sulfoxide reductase msrA/msrB	
			K16787	energy-coupling factor transport system ATP-binding protein	
			K11754	dihydrofolate synthase / folylpolyglutamate synthase	
			K15738	ABC transport system ATP-binding/permease protein	
			K16785	energy-coupling factor transport system permease protein	
			K16798	zinc finger protein GLI2	
			K00527	ribonucleoside-triphosphate reductase (thioredoxin)	
			K06158	ATP-binding cassette, subfamily F, member 3	
			K01154	type I restriction enzyme, S subunit	
	Ottowia_sp._Marseille P4747	2-Chlorohexadecanal Octadecanamide	K16786	energy-coupling factor transport system ATP-binding protein	positively
			K03110	fused signal recognition particle receptor	
			K16787	energy-coupling factor transport system ATP-binding protein	
			K15738	ABC transport system ATP-binding/permease protein	
			K12267	peptide methionine sulfoxide reductase msrA/msrB	
			K16785	energy-coupling factor transport system permease protein	
			K15580	oligopeptide transport system substrate-binding protein	
	Streptococcus_intermedius	Tetracosatetraenoyl carnitine	K00527	ribonucleoside-triphosphate reductase (thioredoxin)	positively
			K01154	type I restriction enzyme, S subunit	
			K15738	ABC transport system ATP-binding/permease protein	
	Strepiococcus_sp._1171_SSPC	Octadecanamide	K03110	fused signal recognition particle receptor	positively
			K16787	energy-coupling factor transport system ATP-binding protein	
	Streptococcus urinalpis	Solutol HS 15	K03110	fused signal recognition particle receptor	positively
gut	Acetobacter malorum	Erucoylacetone	K03336	3D-(3,5/4)-trihydroxycyclohexane-1,2-dione acylhydrolase (decyclizing)	positively

Sinorhizobium meliloti	Erucoylacetone	K03338	5-dehydro-2-deoxygluconokinase	positively
		K03336	3D-(3,5/4)-trihydroxycyclohexane-1,2-dione acylhydrolase (decyclizing)	
		K03338	5-dehydro-2-deoxygluconokinase	
